# Breaking hypoxic barrier: Oxygen-supplied nanomaterials for enhanced T cell-mediated tumor immunotherapy

**DOI:** 10.1016/j.ijpx.2025.100400

**Published:** 2025-09-16

**Authors:** Shuo Xiang, Hui Zhan, Jimin Zhan, Xin Li, Xiaoji Lin, Wenjie Sun

**Affiliations:** a Zhejiang Engineering Research Center for Innovation and Application of Intelligent Radiotherapy Technology, Zhejiang-Hong Kong Precision Theranostics of Thoracic Tumors Joint Laboratory, The Second Affiliated Hospital of Wenzhou Medical University, Wenzhou 325000, China; b College of Materials Science and Engineering, Hunan University, Changsha 410082, China; c Department of Radiation Oncology, The Second Affiliated Hospital and Yuying Children's Hospital of Wenzhou Medical University, Wenzhou 325000, China; d Wenzhou Key Laboratory of Basic Science and Translational Research of Radiation Oncology, The Second Affiliated Hospital of Wenzhou Medical University, Wenzhou 325000, China

**Keywords:** Oxygen-supplied nanomaterials, T lymphocytes, T cell infiltration, T cell response, Hypoxia

## Abstract

Hypoxia in the tumor microenvironment (TME) is a critical barrier to effective cancer immunotherapy, as it suppresses T cell infiltration and response while fostering immune evasion. Oxygen-supplied nanomaterials (OSNs) have recently emerged as promising tools to alleviate hypoxia, modulate the TME, and enhance the efficacy of immunotherapies. This review explores the synergistic interplay between OSNs and T lymphocytes in overcoming hypoxia-driven immune suppression. We discuss the mechanisms by which hypoxia limits T cell functionality, infiltration, and cytotoxicity, and highlight how nanomaterials restore oxygenation, boost immune activation, and improve chemokine-mediated T cell recruitment. Key advances in nanotechnology, including perfluorocarbon-based systems and catalytic nanoparticles, are evaluated for their ability to improve anti-tumor immunity and synergize with immune checkpoint inhibitors and chimeric antigen receptor-T cell therapies. Finally, we address the challenges of nanomaterial delivery, safety, and clinical translation, emphasizing opportunities for personalized strategies. OSNs offer transformative potential to enhance T cell-mediated anti-tumor responses, advancing immunotherapy's frontier.

## Graphical abstract

**Figure:**
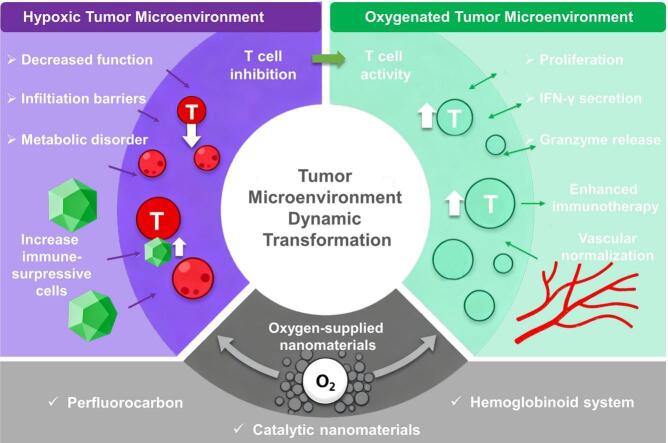

## 1 Introduction

Cancer remains one of leading cause of mortality worldwide, presenting a formidable challenge to healthcare systems (Horgan et al., 2022; Ginsburg et al., 2017; Lai et al., 2025; Li et al., 2024a). Despite considerable progress has been made in conventional treatment modalities such as surgery, radiotherapy, and chemotherapy, many cancers remain resistant to therapy, partly due to the unique and complex nature of the TME (de Visser and Joyce, 2023; Xiao and Yu, 2021). The TME comprises not only cancer cells but also stromal cells, immune cells, blood vessels, signaling molecules, and extracellular matrix (ECM) components, creating a dynamic ecosystem that significantly influences tumor progression and therapeutic outcomes (Wong-Rolle et al., 2020; Bader et al., 2020). Within this microenvironment, hypoxia—characterized by a low oxygen partial pressure—emerges as a hallmark feature of aggressive and treatment-resistant tumors (Jing et al., 2019; Chen et al., 2023a). Hypoxia arises from an imbalance between oxygen supply and demand, driven by rapid tumor growth and aberrant vascularization (Wu et al., 2022a; Chen et al., 2024a). This condition initiates a cascade of adaptive biological responses that promote tumor survival, angiogenesis, metastasis, and immune evasion. Key molecular players, such as hypoxia-inducible factors (HIFs), regulate cellular adaptation to oxygen deprivation, orchestrating gene expression changes that facilitate tumor cell proliferation, migration, and resistance to apoptosis (Bai et al., 2022; Bao and Wong Hypoxia, 2021). Furthermore, hypoxia interferes with effective drug delivery and radiation therapy, compounding the difficulty of treating hypoxic tumors. Therefore, targeting hypoxia and its downstream pathways has emerged as a promising strategy for the development of novel therapeutic strategies.

One of the most detrimental effects of hypoxia is its profound impact on the immune system, particularly on T lymphocytes, which play a pivotal role in anti-tumor immunity (Finisguerra et al., 2023; Zhu et al., 2024a). Cytotoxic T lymphocytes (CTLs), endowed with the ability to recognize and eliminate tumor cells, depend on sufficient oxygen availability to maintain their metabolism, proliferation, and effector functions. Hypoxic conditions suppress T cell infiltration into tumors by disrupting chemokine gradients and downregulating adhesion molecules required for immune cell trafficking (Vignali et al., 2023; Dang et al., 2011). Moreover, hypoxia drives the recruitment and activation of immunosuppressive cells such as regulatory T cells (Tregs) and myeloid-derived suppressor cells (MDSCs), further dampening the anti-tumor immune response. Consequently, tumors in hypoxic environments are often resistant to immunotherapies, including immune checkpoint blockade and adoptive T cell transfer, highlighting the need for innovative solutions to overcome this challenge. T cell infiltration and activation are critical determinants of anti-tumor immunity and are associated with favorable clinical outcome. The presence of tumor-infiltrating T lymphocytes (TILs) has been correlated with improved survival and enhanced response to immunotherapies across various cancers, including melanoma, lung, and colorectal cancer. Effective T cell infiltration requires a well‑oxygenated environment to sustain their metabolic demands and effector functions. Under hypoxia, T cells undergo metabolic reprogramming, shifting from oxidative phosphorylation to glycolysis, which limits their proliferation and cytotoxic potential (Vignali et al., 2023; Scharping et al., 2021). Additionally, hypoxia-induced alterations in the TME, such as increased extracellular acidity and the accumulation of reactive oxygen species (ROS), create a hostile environment that impairs T cell viability and function. Overcoming these barriers is essential to unlock the full potential of T cell-based immunotherapies.

In recent years, nanotechnology has emerged as a transformative field in oncology, offering innovative solutions to address persistent therapeutic challenges (Li et al., 2025; Li et al., 2023; Zhao et al., 2018; Zhou et al., 2018; Li et al., 2017; Li et al., 2021). Among these, Oxygen-supplied nanomaterials (OSNs) have attracted considerable attention for their ability to alleviate hypoxia in the TME. These nanomaterials are engineered to deliver or generate oxygen at the tumor site, restoring normal oxygen levels and mitigating the adverse effects of hypoxia on tumor biology and immune responses (Jiang et al., 2019; Zhang et al., 2022). By reoxygenating the TME, these nanomaterials not only inhibit tumor growth but also potentiate immunotherapy by promoting T cell infiltration and activation (Hatfield et al., 2015; Sitkovsky et al., 2014a). This dual functionality positions OSNs as a promising therapeutic approach in the fight against cancer (Hu et al., 2023).

The fundamental premise that reversing tumor hypoxia can potentiate anti-tumor immunity is strongly supported by a body of pioneering work that laid the conceptual foundation for this field. Seminal studies demonstrated that physiologically relevant hypoxic conditions directly impair T lymphocyte development and effector functions, providing a mechanistic link between low oxygen and immune suppression (Caldwell et al., 2001). The immunostimulatory effects of oxygenation were further elucidated by research showing that systemic oxygenation weakens the hypoxia and HIF-1α-dependent, extracellular adenosine-mediated tumor protection, identifying a key immunosuppressive pathway that is disrupted by increased oxygen availability (Hatfield et al., 2014). This established the concept of the “hostile, hypoxia–A2-adenosinergic” tumor biology as a major barrier to immunotherapy, which must be overcome for successful treatment (Sitkovsky et al., 2014b). Crucially, direct experimental evidence confirmed the immunological mechanisms of the antitumor effects of supplemental oxygenation, demonstrating that breathing high levels of oxygen (hyperoxia) can reduce hypoxia, decrease levels of immunosuppressive molecules like adenosine, and enhance the efficacy of adoptive T cell therapy in mouse models (Hatfield et al., 2015). Subsequent work expanded this concept, outlining how oxygenation can improve cancer vaccines, adoptive cell transfer, and blockade of immunological negative regulators (Hatfield and Sitkovsky, 2015), and reviewing the potential of antihypoxic oxygenation agents with respiratory hyperoxia to improve cancer immunotherapy (Hatfield and Sitkovsky, 2020a). Most recently, this principle was advanced using nanotechnology, showing that oxygen-carrying nanoemulsions combined with respiratory hyperoxia can eliminate tumor hypoxia–induced immunosuppression and enhance checkpoint blockade therapy (Halpin-Veszeleiova et al., 2025). These foundational studies collectively provide the critical rationale and mechanistic underpinnings for the development of oxygen-supplying nanomaterials, positioning them as a targeted strategy to achieve the immunologically beneficial effects of tumor reoxygenation.

This review aims to explore the synergistic relationship between OSNs and T lymphocytes in the context of cancer immunotherapy. Specifically, we will examine how these nanomaterials can enhance T cell functionality, improve tumor infiltration, and potentiate anti-tumor immune responses. The discussion begins with an overview of the mechanisms by which hypoxia suppresses T cell activity and contributes to immune evasion in the TME. Next, wedelve into the design, mechanisms, and applications of OSNs, emphasizing their potential to overcome hypoxia-induced barriers to immunotherapy. The review also explores how these nanomaterials interact with T lymphocytes, both directly and indirectly, to enhance their cytotoxic activity and promote tumor infiltration. This includesthe effects of reoxygenation on T cell metabolism, proliferation, and effector functions, as well as the modulation of chemokine gradients and vascular activation to facilitate T cell trafficking. Furthermore, we discuss preclinical and clinical studies that demonstrate the efficacy of OSNs in improving the outcomes of immune checkpoint blockade, adoptive T cell therapies, and cancer vaccines.

Finally, the review addresses the challenges and opportunities associated with the clinical translation of OSNs. While these nanomaterials hold immense promise, their successful integration into clinical practice requires overcoming technical hurdles related to delivery, stability, and safety. Additionally, there is a need for personalized approaches to account for the heterogeneity of tumor biology and the TME across patients. By synthesizing current knowledge and identifying gaps in the field, this review seeks to provide a comprehensive framework to guide future research and development at the intersection of nanotechnology and immunotherapy. In conclusion, the convergence of OSNs and T lymphocyte-based immunotherapies represents a transformative approach in cancer treatment. By simultaneously addressing the dual challenges of hypoxia and immune suppression in the TME, these strategies offer the potential to substantially improve patient outcomes and advance the frontiers of oncology. This review serves as a timely and comprehensive assessment of the potential of these cutting-edge technologies to revolutionize cancer immunotherapy and pave the way for novel therapeutic paradigms.

## 2 The role of hypoxia in TME and immune evasion

The TME is a highly dynamic and complex ecosystem that significantly influences cancer progression and treatment outcomes. Among the hallmarks of the TME, hypoxia—characterized by reduced oxygen tension—is a central factor driving tumor aggressiveness and immune evasion (Bai et al., 2022; Yuan et al., 2021). Hypoxia arises results from an imbalance between the rapid growth of tumor cells and the limited oxygen supply provided by abnormal vasculature. This oxygen deprivation profoundly impacts tumor biology and immune responses, fostering a microenvironment that promotes tumor survival while suppressing anti-tumor immunity (Wang et al., 2024; Dekker et al., 2022).

### 2.1 Impact of hypoxia on tumor biology

**Fig. 1:**
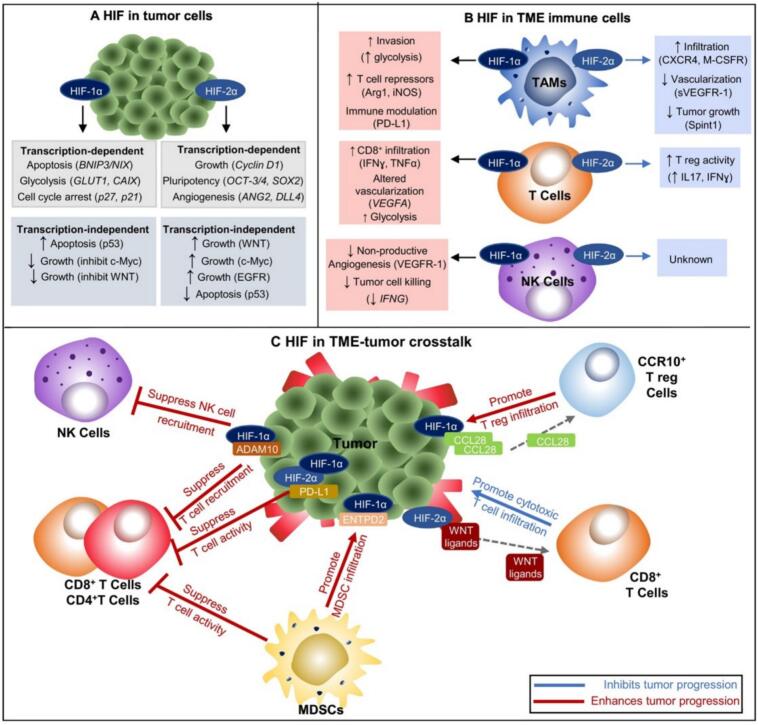
Multifaceted Roles of HIF Isoforms in Tumors and the Immune Microenvironment. (A) Tumor Cell-Specific Roles: HIF-1α and HIF-2α regulate distinct transcription-dependent and -independent targets (excluding shared ones). Their activation can promote or suppress tumors depending on context. (B) Immune Cell-Specific Roles: In the TME, HIF-1α and HIF-2α exert distinct, non-overlapping effects on immune cell activation, influencing tumor progression. (C) Tumor–Immune Crosstalk: Tumor-specific HIF expression reshapes tumor–immune cell interactions, affecting immune cytotoxicity and responses to therapy. (Reprint with permission from Ref. (Cowman and Koh, 2022).)

Hypoxia profoundly alters in tumor behavior, primarily through the activation of HIFs, a family of transcription factors that regulate cellular responses to oxygen deprivation (Fig.1) (Cowman and Koh, 2022). HIF-1α and HIF-2α are the most studied isoforms and play pivotal roles in tumor adaptation to hypoxia (Cowman and Koh, 2022; Prabhakar et al., 2020). Firstly, hypoxia drives angiogenesis, the formation of new blood vessels, by upregulating vascular endothelial growth factor (VEGF) (Florentin et al., 2022; Han et al., 2024). While angiogenesis is essential for supplying tumors with nutrients and oxygen, the newly formed vasculature is often abnormal, characterized by leakiness and structural disorganization. These defective blood vessels fail to alleviate hypoxia, perpetuating a cycle of oxygen deprivation and aberrant vascularization. Secondly, hypoxia promotes metabolic reprogramming in tumor cells. Under oxygen-deprived conditions, cancer cells shift from oxidative phosphorylation to glycolysis, a phenomenon known as the Warburg effect (Vaupel et al., 2019; Icard et al., 2018). This metabolic adaptation allows tumors to generate energy even in low-oxygen environments. However, it also leads to the accumulation of lactate and acidification of the extracellular matrix ECM, fostering a hostile microenvironment that impairs immune cell function.

Moreover, hypoxia enhances the metastatic potential of tumors by facilitating epithelial-to-mesenchymal transition (EMT). This process enables cancer cells to lose their epithelial characteristics, gain migratory and invasive capabilities, and disseminate to distant tissues. Hypoxia also activates matrix metalloproteinases (MMPs), which degrade the ECM, further promoting invasion and metastasis (Tong and Zhang, 2012; Zhou et al., 2024). Additionally, hypoxia contributes to resistance against conventional therapies such as chemotherapy and radiotherapy by activating survival pathways in tumor cells and impairing drug and oxygen delivery.

### 2.2 Hypoxia-induced suppression of immune cell function

**Fig. 2:**
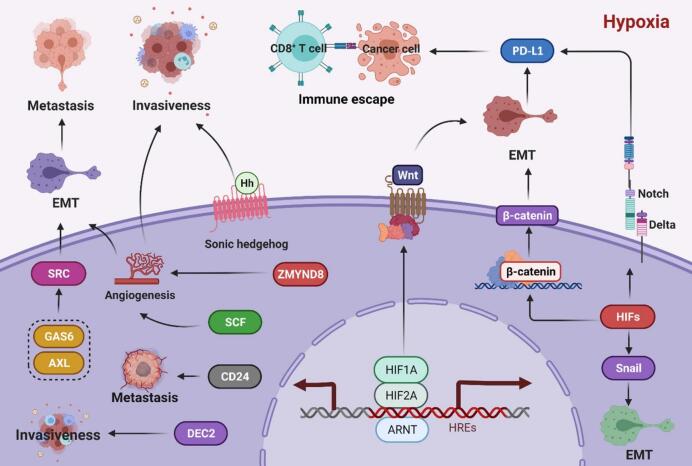
Role of HIFs in Cancer Invasiveness and Metastasis. (Reprint with permission from Ref. (Wu et al., 2022a).)

Hypoxia is a major driver of immune suppression in the TME, creating an environment that impairs the function and recruitment of anti-tumor immune cells while favoring immunosuppressive populations (Fig. 2) (Wu et al., 2022a). Effector immune cells, such as CTLs and helper T cells, are particularly vulnerable to the detrimental effects of hypoxia (Sattiraju et al., 2023; Palazon et al., 2017). These cells depend on adequate oxygen availability to support energy-intensive processes, including proliferation, cytokine production, and cytotoxic activity. In a hypoxic TME, T cells undergo metabolic dysfunction, which compromises their ability to eliminate tumor cells. In addition to directly impairing effector T cells, hypoxia promotes the accumulation of immunosuppressive cells, such as Tregs and MDSCs. These cells inhibit CTLs and natural killer (NK) cells activity by secreting immunosuppressive cytokines, such as IL-10 and TGF-β, and expressing checkpoint molecules like programmed death-ligand 1 (PD-L1) (Zalfa and Paust, 2021). Hypoxia also disrupts the function of antigen-presenting cells, such as dendritic cells (DCs), which are critical for initiating T cell responses (Qu et al., 2023; Fan et al., 2022; Han et al., 2021a). In hypoxic conditions, DC maturation and antigen presentation are impaired, leading to suboptimal T cell activation. Additionally, hypoxia polarizes macrophages toward an M2-like phenotype, which is associated with tissue remodeling and immune suppression. These tumor-associated macrophages (TAMs) secrete VEGF and immunosuppressive cytokines, thereby reinforcing the immunosuppressive nature of the TME (Bai et al., 2022; Vitale et al., 2019).

HIF1A enhances cancer cell invasiveness by activating the Sonic Hedgehog pathway and upregulating DEC2, which further amplifies HIF1A activity, driving metabolic reprogramming, angiogenesis, and invasion. HIF2A promotes SCF transcription via hypoxia-responsive elements (HREs), supporting angiogenesis and metastasis. Both HIF1A and HIF2A target ZMYND8, which boosts angiogenesis and reduces cell death in breast cancer. They also activate the GAS6/AXL pathway, triggering SRC and MET signaling to regulate EMT and metastasis. HIF1A induces CD24 expression, fostering tumor growth and spread. Hypoxia-driven HIFs promote EMT through Snail, β-catenin, Wnt, and Notch pathways, enhancing metastasis, tumor survival, and immune evasion via PD-L1 upregulation (Wu et al., 2022a).

A pivotal mediator of hypoxia-induced immunosuppression is the elevated expression of transforming growth factor-beta 1 (TGF-β1) within the TME. Hypoxia stabilizes HIF-1α, which in turn can directly or indirectly promote the transcription and activation of TGF-β1 (Bandopadhyay and Patranabis, 2023; Copple, 2010). This potent immunosuppressive cytokine establishes a formidable barrier to anti-tumor immunity through multiple mechanisms. Crucially, TGF-β1 plays a master role in driving the differentiation of naïve CD4^+^ T cells into FoxP3^+^ Tregs (Chen et al., 2003), thereby expanding a population dedicated to suppressing effector T cell responses. Furthermore, TGF-β1 directly impairs the function of CTLs by suppressing the expression of key effector molecules, most notably granzyme B and perforin, which are essential for tumor cell killing (Thomas and Massagué, 2005; Yang et al., 2010). It also inhibits the expression of cytolytic proteins and interferon-gamma (IFN-γ) production, effectively crippling the CTLs' offensive capabilities. Thus, the hypoxia-HIF-TGF-β axis constitutes a central mechanism by which the TME fosters immune tolerance and facilitates tumor escape. Targeting this axis, potentially through the use of OSNs to alleviate the hypoxic driver, represents a promising strategy to reverse TGF-β-mediated immunosuppression and restore effective anti-tumor immunity.

### 2.3 Mechanisms of cellular adaptation to hypoxia in tumor and immune cells

**Fig. 3:**
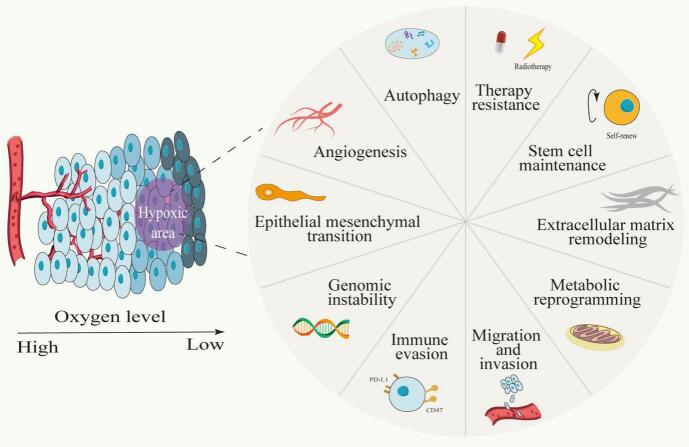
Overview of the effects of hypoxia on tumor cells. (Reprint with permission from Ref. (Bai et al., 2022)).

Hypoxia induces a range of adaptive responses in both tumor and immune cells, primarily orchestrated by HIFs (Fig. 3) (Bai et al., 2022). In tumor cells, HIF-1α and HIF-2α activation promotes metabolic reprogramming toward glycolysis (the Warburg effect), enhances angiogenesis through VEGF upregulation, and facilitates cell adhesion and invasion via integrins and MMPs (Bai et al., 2022; Bao and Wong Hypoxia, 2021; Vaupel et al., 2019; Icard et al., 2018). These adaptations not only support tumor survival and progression but also contribute to therapy resistance.

Immune cells infiltrating the hypoxic TME also employ HIF-mediated adaptive mechanisms. For instance, DCs and macrophages upregulate HIF-1α to enhance glycolytic metabolism and maintain functionality under low oxygen (Qu et al., 2023; Han et al., 2021a). However, tumors often exploit these pathways to evade immune surveillance. One key strategy involves the accumulation of immunosuppressive metabolites such as L-kynurenine, a tryptophan derivative produced by indoleamine 2,3-dioxygenase (IDO) expressed in tumor and stromal cells. L-kynurenine suppresses T cell function by promoting Treg differentiation and inhibiting CTL activity (Munn and Mellor, 2016; Schlichtner et al., 2023). Additionally, tumor cells exhibit heightened glucose uptake via overexpression of glucose transporters (e.g., GLUT1), leading to glucose deprivation in the TME. This metabolic competition starves infiltrating T cells, impairing their glycolytic metabolism—a process essential for effector function and proliferation (Vignali et al., 2023; Scharping et al., 2021). Consequently, even when immune cells successfully enter the TME, their anti-tumor capacity is severely compromised by these hypoxia-driven metabolic constraints.

Understanding these dual adaptation mechanisms—both within tumor and immune cells—highlights the complexity of targeting hypoxia in cancer therapy. Strategies that combine OSNs with inhibitors of IDO or glucose metabolism may offer synergistic benefits in restoring anti-tumor immunity.

### 2.4 Impaired T cell infiltration and response

**Fig. 4:**
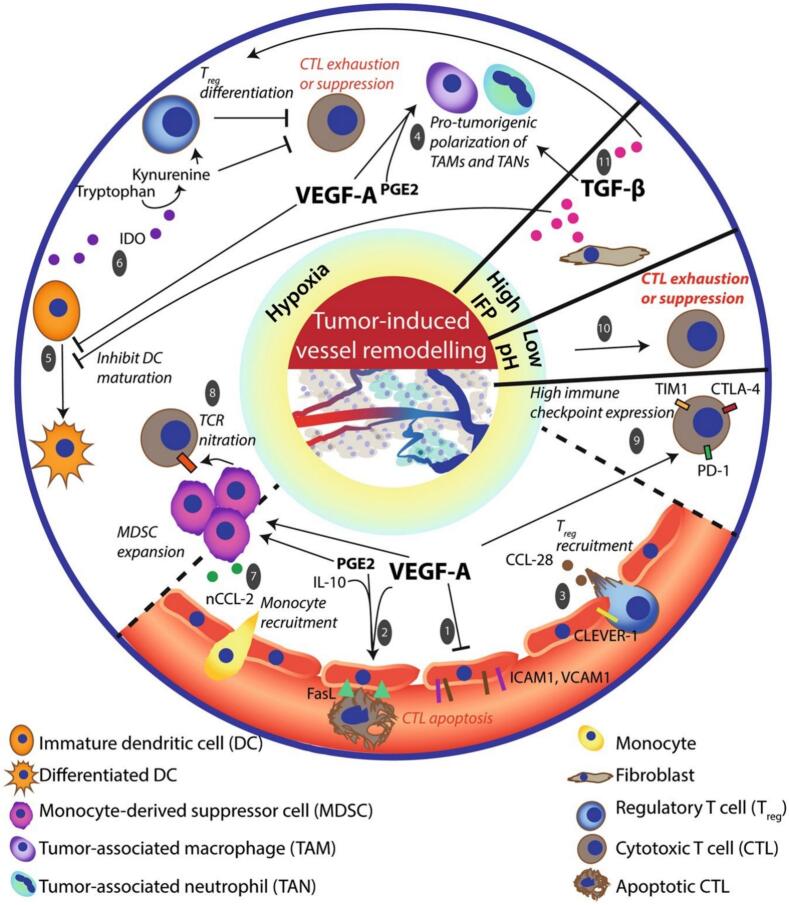
The nature of the TME influences immune cell composition and hampers antitumor immunity. (Reprint with permission from Ref. (Schaaf et al., 2018).)

T cell infiltration into tumors is a critical determinant of effective anti-tumor immunity. However, hypoxia poses substantial significant barriers to T cell trafficking and functionality (Fig. 4) (Schaaf et al., 2018).

A fundamental reason for the lack of T cell infiltration is that lymphocytes actively avoid trafficking into severely hypoxic regions. This phenomenon is not merely a passive consequence of dysfunctional vasculature but also an active process mediated by hypoxia-induced immunosuppressive pathways. As elucidated in the pivotal study by Hatfield et al., the accumulation of extracellular adenosine, a key immunosuppressive metabolite whose production is dramatically enhanced in hypoxia via HIF-1α-dependent upregulation of CD73 and CD39, creates a chemical barrier that inhibits the migration and function of CTLs (Hatfield et al., 2015). This adenosine-rich microenvironment effectively signals lymphocytes to avoid the hypoxic tumor core, representing a critical mechanism of immune evasion that must be overcome for successful therapy. Chemokines, which guide T cells to the tumor site, are often downregulated under hypoxic conditions. For example, CXCL9 and CXCL10, key chemokines that attract CTLs, are suppressed under hypoxic conditions (Hatfield et al., 2015; Su et al., 2024; Cunningham et al., 2022). Additionally, hypoxia reduces the expression of chemokine receptors on T cells, further impairing their ability to migrate toward tumors (Korbecki et al., 2021). Hypoxia-induced the abnormal vasculature also hampers T cell infiltration. Tumor blood vessels often lack proper endothelial junctions and adhesion molecules, such as intercellular adhesion molecule-1 (ICAM-1) and vascular cell adhesion molecule-1 (VCAM-1), which are essential for T cell extravasation (Takasawa et al., 2024; Kawczyk-Krupka et al., 2017). Without these molecules, T cells cannot efficiently migrate from the bloodstream into the tumor tissue. Once inside the hypoxic TME, T cells encounter additional challenges. The metabolic competition between tumor cells and immune cells for glucose and other nutrients depletes the resources necessary for T cell proliferation and effector function. The acidic and oxidative conditions of the TME further inhibit T cell activity by disrupting signaling pathways and inducing apoptosis.

### 2.5 Role of HIFs in immune suppression

HIFs are master regulators of the hypoxic response and play a central role in mediating the immunosuppressive effects of hypoxia. In T cells, HIF-1α diminishes cytotoxic activity by promoting metabolic reprogramming (Bao and Wong Hypoxia, 2021; Seo et al., 2022; Sitkovsky and Lukashev, 2005). This shift from oxidative phosphorylation to glycolysis reduces ATP production, which is critical for T cell functionality. HIF-1α also upregulates immune checkpoint molecules such as PD-L1 on tumor cells, directly inhibiting T cell activity (Noman et al., 2014). HIFs also contribute to the maintenance and expansion of immunosuppressive populations in the TME. For example, HIF-1α enhances the stability and function of Tregs by upregulating FoxP3, a transcription factor critical for Treg activity (Hatfield et al., 2015; Abdelhafiz et al., 2021; Chen et al., 2023b). HIF-1α also promotes the recruitment and activation of MDSCs, which suppress T cell responses through arginase-1 activity and the production of ROS. In macrophages, HIFs drive polarization toward the M2-like phenotype, which supports tumor growth and immune suppression. These macrophages secrete VEGF, IL-10, and other factors, further exacerbating vascular abnormalities and promoting immune evasion (Wu et al., 2022a). Specifically, under normal oxygen, HIF-1α is degrade, making tumor cells vulnerable to immune surveillance. In hypoxia, HIF-1α evades degradation, translocates to the nucleus, and dimerizes with ARNT which upregulates PD-L1and inducing CTL apoptosi. HIF-1α also impairs NO signaling, increases tumor cell-platelet interactions, regulates CD47 expression to prevent phagocytosis and drives VEGF-mediated angiogenesis, facilitating metastasis and immune escape (Wu et al., 2022a).

Beyond the well-established hypoxia-PD-L1 axis, another crucial immune checkpoint, VISTA (V-domain immunoglobulin-containing suppressor of T cell activation), has been identified as a direct transcriptional target of HIF-1α (Deng et al., 2019; Abooali et al., 2025). VISTA is primarily expressed on myeloid cells and a subset of T cells within the TME, where it functions as a potent negative regulator of T cell activation and proliferation. Its mechanism involves the inhibition of T cell receptor (TCR) signaling and the promotion of the development of Tregs (Rohatgi and Kirkwood, 2021).

Under hypoxic conditions, HIF-1α binds directly to HREs in the promoter region of the VSIR gene (encoding VISTA), leading to its transcriptional upregulation (Deng et al., 2019). This HIF-1α-driven VISTA expression represents a fundamental mechanism by which the immunosuppressive TME is further amplified. The induction of VISTa adds another layer of immune resistance, complementing the PD-L1/PD-1 pathway and contributing to T cell exhaustion and dysfunction (Abooali et al., 2025). Consequently, the hypoxic TME co-opts multiple, synergistic checkpoint pathways to evade immune destruction. Targeting VISTA, either alone or in combination with other checkpoints like PD-1/PD-L1, has emerged as a promising therapeutic strategy, particularly in tumors with significant hypoxia (Rohatgi and Kirkwood, 2021). The inclusion of VISTA in the spectrum of hypoxia-regulated immune checkpoints underscores the complexity of the immunosuppressive network in the TME and highlights the potential of OSNs to modulate a broader range of immune evasion mechanisms.

Furthermore, HIF-1α plays a pivotal role in establishing the potent immunosuppressive hypoxia-adenosinergic pathway within the TME. It directly promotes the transcription of ectonucleotidases CD39 and CD73 on the surface of tumor and stromal cells. These enzymes catalyze the sequential hydrolysis of extracellular ATP to adenosine, leading to the accumulation of this immunosuppressive metabolite in hypoxic regions (Sitkovsky et al., 2004; Eltzschig and Carmeliet, 2011). The foundational importance of this pathway is underscored by two seminal works. First, it was established that A2A adenosine receptor signaling acts as a general negative regulator of inflammation and tissue damage, providing a physiological mechanism to protect tissues from excessive immune responses (Ohta and Sitkovsky, 2001). Tumors co-opt this protective mechanism for immune evasion. This was conclusively demonstrated by Ohta et al., who showed that the A2A adenosine receptor protects tumors from antitumor T cells. Genetic deletion or pharmacological blockade of the A2A receptor on T cells broke their tolerance to tumors and elicited potent anti-tumor immune responses in mouse models, establishing tumor-derived adenosine as a key mediator of immune escape (Ohta et al., 2006). Thus, the HIF-1α-CD39/CD73-A2AR axis constitutes a critical immunosuppressive circuit in the hypoxic TME, and targeting it represents a promising therapeutic strategy.

## 3 OSNs: an overview

The development of OSNs has brought significant advancements in cancer therapy, particularly in overcoming the challenges associated with tumor hypoxia (Zhang et al., 2022; Huang et al., 2022). Hypoxia, or low oxygen availability, is a defining characteristic of the TME that impairs immune responses and limits the efficacy of therapies, including immunotherapy. OSNs are engineered to alleviate hypoxia by delivering or generating oxygen within the TME, creating a more favorable environment for therapeutic interventions (Wu et al., 2022b). This section elaborates on the types, mechanisms, and advantages of these innovative materials in cancer immunotherapy.

### 3.1 Definition and types

OSNs are nanoscale systems engineered to alleviate hypoxia in the TME, either by delivering oxygen directly or by generating oxygen in situ. Several types of nanomaterials have been developed, each utilizing distinct strategies to address hypoxia. One prominent class is perfluorocarbon (PFC)-based materials, which exploit the exceptional oxygen-carrying capacity of PFCs (Yang et al., 2022).Chemically inert and highly efficient at dissolving and transporting oxygen, PFCs are typically formulated into nanoparticles with surfactant coatings or encapsulated within liposomes to enhance stability and prolong circulation in the bloodstream. Once administered, PFC nanoparticles transport oxygen from the lungs to the hypoxic regions of tumors, replenishing oxygen levels (Krafft, 2020). Another notable category includes catalase-loaded nanoparticles, which use the enzyme catalase to convert hydrogen peroxide (H₂O₂) into oxygen and water (Heble et al., 2021; Zhang et al., 2017). Tumors typically exhibit high levels of H₂O₂ due to their altered metabolism. Catalase-loaded nanoparticles exploit this unique feature of the TME to generate oxygen locally, alleviating hypoxia without requiring external oxygen sources (Song et al., 2016; Hu et al., 2018). These nanoparticles are often fabricated using biocompatible and biodegradable polymers or lipid-based carriers.

**Fig. 5:**
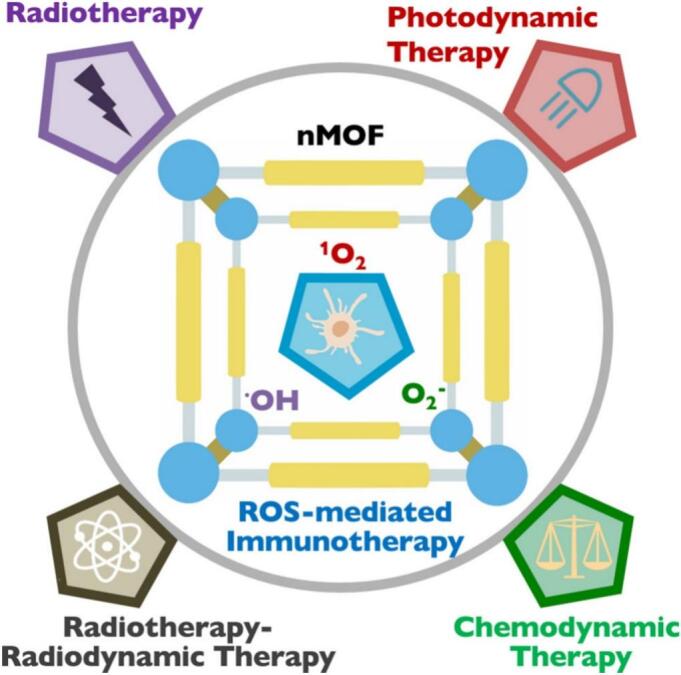
A schematic illustrating the mechanisms of local nMOF-mediated therapies, including photodynamic therapy, radiotherapy, radiodynamic therapy, and chemodynamic therapy, highlighting their role in promoting ROS generation. This ROS production effectively kills tumor cells and induces localized inflammation, thereby enhancing both innate and adaptive immune responses. (Reprint with permission from Ref. (Ni et al., 2020a).)

Hemoglobin-mimicking systems represent another innovative class of OSNs (Sun et al., 2024). These systems are designed to replicate the oxygen-binding and -transporting functions of natural hemoglobin. Engineered hemoglobin-mimicking nanoparticles can reversibly bind oxygen and release it in response to hypoxic conditions. Protective coatings are often applied to improve stability and minimize immune recognition and prolong circulation time, thereby improving therapeutic efficacy. Metal-organic frameworks (MOFs) are porous, crystalline materials composed of metal ions coordinated with organic ligands (Wu and Yang, 2017; Xu et al., 2021). MOFs can encapsulate and release oxygen molecules or serve as catalysts to generate oxygen from endogenous H₂O₂ (Fig. 5) (Xu et al., 2021; Ni et al., 2020a). Their tunable structures and high surface areas make them particularly versatile for oxygen delivery applications. Another emerging strategy involves photosensitizer-integrated nanomaterials, which release oxygen or generate ROS upon light exposure. These materials are especially useful in photodynamic therapy (PDT), where light activation enhances tumor oxygenation and ROS production, amplifying the anti-tumor effect.

### 3.2 Mechanisms of action

**Fig. 6:**
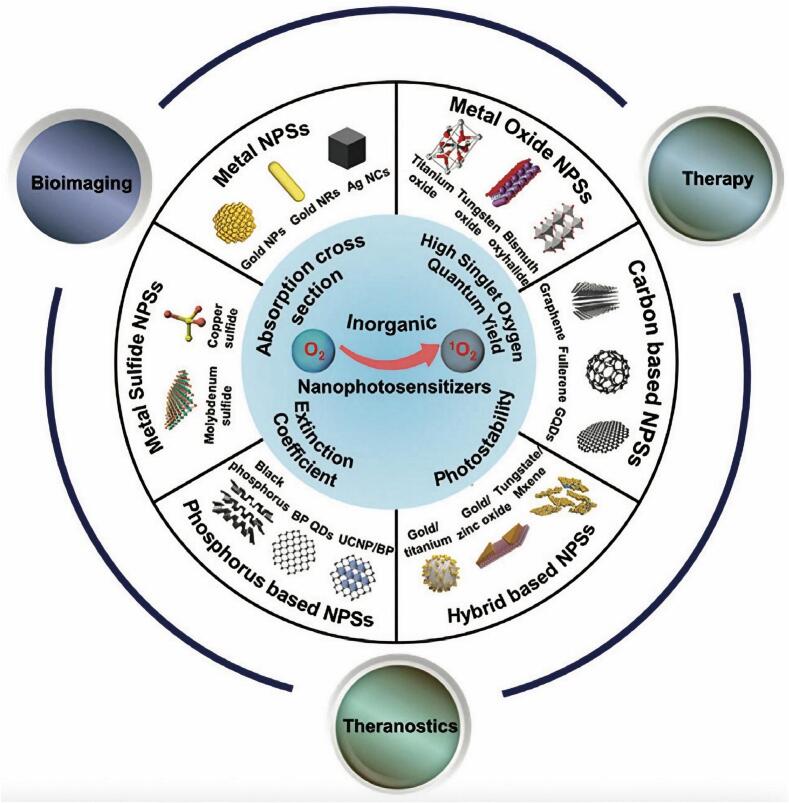
Representation of a wide variety of inorganic NPSs with intrinsic ^1^O_2_ generation capacity for PDT as well as their potential for tumor imaging, therapy, and multimodal imaging guided synergistic therapy. (Reprint with permission from Ref. (Younis et al., 2021).)

The mechanisms by which OSNs function depend on their ability to deliver oxygen directly or generate it through biochemical reactions (Fig. 6) (Younis et al., 2021). These processes rely on targeted tumor delivery, controlled oxygen release, and interaction with the unique features of the TME. Direct oxygen delivery is a primary mechanism employed by PFC-based systems and hemoglobin-mimicking nanoparticles (Yang et al., 2023a; Jägers et al., 2021). These materials dissolve or bind oxygen molecules in the bloodstream and selectively release them in hypoxic tumor regions. This approach effectively restores oxygen levels, mitigating the adverse effects of hypoxia. In contrast, oxygen generation involves chemical reactions within the TME to produce oxygen in situ. Catalase-loaded nanoparticles and certain MOFs capitalize on the elevated H₂O₂ levels in tumors, catalyzing its decomposition into oxygen and water. This not only alleviates hypoxia but also reduces oxidative stress, which can contribute to tumor progression.

Another critical aspect of these nanomaterials is their biodegradation and safety profiles. Most OSNs are constructed from biocompatible and biodegradable components, such as natural polymers, lipids, and proteins. This ensures that the materials break down into non-toxic byproducts after fulfilling their therapeutic function. For metal-based systems, such as MOFs, surface modifications with biocompatible coatings minimize toxicity and facilitate safe clearance from the body. To enhance specificity, OSNs often incorporate targeted delivery mechanisms. These may include ligands that recognize tumor-specific markers or pH-sensitive coatings that activate in the acidic conditions of the TME. In some cases, external stimuli such as light, ultrasound, or magnetic fields are used to trigger oxygen release at the tumor site, reducing off-target effects.

The ultimate therapeutic efficacy of these platforms is contingent upon their ability to significantly elevate intratumoral oxygen levels (pO₂). A quantitative comparison of their oxygenation performance, as reported in preclinical studies, provides critical insights for platform selection. PFC-based systems typically demonstrate a rapid but often transient increase in pO₂, with studies reporting boosts from severe hypoxia (<10 mmHg) to high level (≈107 mmHg) shortly after administration, leveraging their high oxygen-dissolving capacity (Halpin-Veszeleiova et al., 2025). In contrast, catalase-loaded or catalytic nanomaterials can achieve more sustained oxygenation by continuously generating O₂ from endogenous H₂O₂. Their efficacy is highly dependent on the local H₂O₂ concentration, with successful systems reporting substantial pO₂ increases from ∼5 mmHg to over 60 mmHg, effectively reversing hypoxia for extended periods (Heble et al., 2021). MOFs with oxygen-carrying or -generating capabilities can show high performance due to their immense surface area, with some designs achieving a greater than 5-fold increase in pO₂, pushing levels from severely hypoxic (e.g., 5 mmHg) to well-oxygenated states (25 mmHg) (Kong et al., 2025). It is important to note that direct comparisons should be made with caution due to variations in measurement techniques, tumor models, and dosing regimens across studies.

This quantitative perspective informs their potential application. For instance, catalytic nanomaterials might be uniquely suited for treating “hot” but hypoxic tumors characterized by high immune infiltration but suppressed function, as their ability to provide sustained oxygenation and simultaneously reduce oxidative stress can directly ameliorate the metabolic barriers to T cell activity. In contrast, PFCs or hemoglobin-based systems, with their rapid oxygen release, might be ideal for acutely enhancing the efficacy of oxygen-dependent therapies like radiotherapy in “cold” tumors, where the primary goal is to rapidly sensitize a large volume of tumor cells to treatment.

### 3.3 Comparative analysis of OSNs

**Fig. 7:**
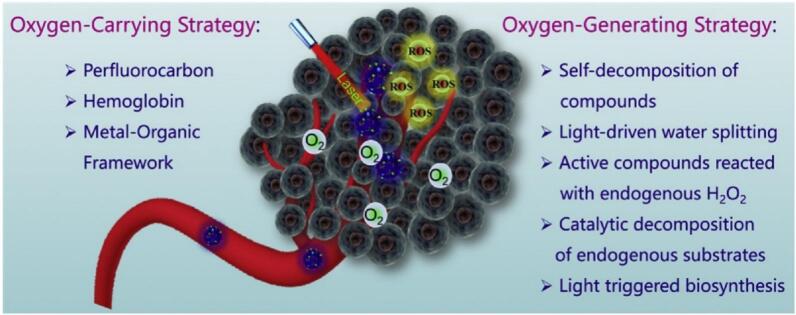
Nanomaterials-mediated oxygenation strategies for enhanced PDT. (Reprint with permission from Ref. (Zhang et al., 2020).)

The distinct compositions and mechanisms of action of various OSNs confer upon them unique advantages and limitations, making them differentially suited for specific therapeutic contexts (Fig. 7) (Zhang et al., 2020). A critical comparative analysis is essential for their rational selection and application.

PFC-based nanomaterials function primarily through the physical dissolution and delivery of oxygen. Their key advantages include high oxygen-carrying capacity, chemical inertness, and biocompatibility, alongside tunable surface chemistry for functionalization (Lambert et al., 2019). However, their passive oxygen release may lack spatiotemporal control, and they pose potential risks of long-term retention in the reticuloendothelial system (RES) organs, such as the liver and spleen (Chen et al., 2013). Their efficacy also inherently depends on adequate systemic oxygenation. Thus, PFCs are ideally deployed in moderately hypoxic tumors with relatively well-perfused areas, often as adjuvants to enhance oxygen-dependent therapies like radiotherapy or for real-time oxygenation imaging (Song et al., 2017).

In contrast, catalase-loaded or catalytic nanomaterials generate oxygen in situ through the catalytic decomposition of endogenous H₂O₂. This self-supplying mechanism, independent of external oxygen sources, is a significant advantage, as it simultaneously reduces oxidative stress by consuming H₂O₂ and offers high catalytic efficiency (Munjal and Dutta, 2021). Their primary limitation lies in their dependence on highly variable and often heterogeneous intratumoral H₂O₂ levels. Additional challenges include ensuring enzyme stability and preventing leakage under biological conditions, as well as the potential immunogenicity of foreign enzymes. These systems are particularly well-suited for treating tumors characterized by high H₂O₂ production, such as highly metabolic or inflamed tumors, and are ideal for combination with therapies that induce ROS (Hu et al., 2018).

Hemoglobin-mimicking nanomaterials operate via the reversible binding and release of oxygen, mimicking natural hemoglobin (Jia et al., 2016; Jansman et al., 2020). Their biomimetic nature allows for high-affinity oxygen binding and the potential for stimuli-responsive release triggered by factors like pH or hypoxia. The main challenges associated with these systems include the risk of iron-induced oxidative toxicity and ROS generation, potential immunogenicity, and the requirement for sophisticated coatings to prevent premature oxidation into non-functional methemoglobin (Jansman et al., 2020; Jahr et al., 2011). These materials are best applied in scenarios requiring sustained and controlled oxygen release within deeply hypoxic tumor cores, where their biomimetic kinetics are most advantageous.

Finally, MOFs can either store/release oxygen or catalytically generate it from H₂O₂. Their unparalleled advantages include extremely high surface area and porosity for large oxygen payloads, highly tunable structure and functionality, and the capacity to integrate multiple therapeutic agents (e.g., drugs, photosensitizers) for combination therapy (Ni et al., 2020b). Their limitations, however, involve the potential toxicity of metal ions upon degradation, challenges regarding stability in physiological conditions, and the complexity of synthesis and scale-up. MOFs are ideally positioned as versatile, multifunctional theranostic platforms for precise, stimuli-responsive delivery and for applications requiring a combination of oxygen delivery with imaging or other therapeutic modalities (Ni et al., 2020b).

In conclusion, the selection of an optimal OSN is not one-size-fits-all but must be guided by a careful consideration of the target tumor's specific pathophysiology (e.g., the degree and heterogeneity of hypoxia, levels of H₂O₂, and vascularization) and the nature of the intended combination therapy. This comparative understanding is paramount for the rational design and successful clinical translation of these innovative nanotechnologies.

### 3.4 Advantages in cancer immunotherapy

OSNs hold transformative potential for cancer immunotherapy by addressing the immunosuppressive effects of hypoxia in the TME (Hatfield and Sitkovsky, 2020a; Halpin-Veszeleiova et al., 2025). Their ability to reoxygenate tumors offers several critical advantages that enhance the efficacy of immunotherapies. One key benefit is the restoration of T cell functionality. Hypoxia suppresses CTLs by disrupting their metabolism, proliferation, and cytokine production (Hatfield et al., 2015; Hatfield and Sitkovsky, 2020b). By reoxygenating the TME, OSNs can reverses these effects, allowing T cells to regain their effector functions and mount a robust anti-tumor response. Another major advantage is the enhancement of T cell infiltration. Hypoxia impairs T cell recruitment to tumors by disrupting chemokine gradients and downregulating adhesion molecules on tumor vasculature. By alleviating hypoxia-induced vascular abnormalities, OSNs restore chemokine signaling and enable CTLs to infiltrate tumors more effectively (Hatfield et al., 2015; Sitkovsky et al., 2014a; Hatfield et al., 2014; Hatfield and Sitkovsky, 2015; Hatfield and Sitkovsky, 2020a; Halpin-Veszeleiova et al., 2025).

OSNs also show significant synergy with immune checkpoint inhibitors (Hatfield et al., 2015; Hatfield and Sitkovsky, 2020a; Halpin-Veszeleiova et al., 2025; Tittarelli et al., 2020; Yin et al., 2023). Checkpoint blockade therapies, such as anti-PD-1 and anti-CTLA-4 antibodies, are designed to reinvigorate exhausted T cells. However, their efficacy is often limited in hypoxic tumors due to insufficient T cell infiltration and activation. By reoxygenating the TME, OSNs enhance T cell activity and improve the response to checkpoint inhibitors. Furthermore, these nanomaterials help reduce immunosuppressive cell populations in the TME. Hypoxia promotes the accumulation of Tregs and MDSCs, which suppress anti-tumor immunity. OSNs reprogram the TME by decreasing these populations, creating a more favorable environment for immune activation (Hatfield et al., 2015; Hatfield and Sitkovsky, 2020a; Halpin-Veszeleiova et al., 2025). Finally, OSNs enable improved combination therapies. For example, integrating these materials with PDT enhances ROS production, amplifying tumor cell death and immune activation (Hou et al., 2020a; Yi et al., 2020). Similarly, pairing OSNs with adoptive T cell therapy or cancer vaccines boosts overall anti-tumor immunity, providing a multifaceted approach to cancer treatment (Hatfield and Sitkovsky, 2015; Hatfield and Sitkovsky, 2020a; Halpin-Veszeleiova et al., 2025).

The versatility of nanoplatforms extends far beyond oxygen delivery, offering innovative strategies to reprogram the immunosuppressive microenvironment and address critical clinical challenges such as metastasis and postoperative recurrence. A series of groundbreaking studies exemplify this by designing multifunctional systems that integrate catalytic therapy with antigen capture and immune modulation. For instance, a versatile catalytic dual oxide antigen-captured nanosponge has been developed to reprogram dysfunctional DCs in the premetastatic niche. This system not only exerts catalytic activity to remodel the immunosuppressive microenvironment but also efficiently captures and retains tumor-associated antigens, subsequently enhancing their uptake and presentation by DCs, thereby remotely enhancing immunotherapy against lung metastasis (Chiang et al., 2025). Addressing the formidable challenge of postoperative brain tumor recurrence, a self-cascading catalytic therapy and antigen capture scaffold has been engineered. This implantable scaffold locally generates ROS through a catalytic cascade to eliminate residual tumor cells, while simultaneously capturing released tumor antigens in situ. This dual action promotes robust antigen presentation and T cell priming directly at the surgical cavity, effectively augmenting postoperative brain immunotherapy (Yalamandala et al., 2025). Complementing this, a novel approach utilizing near-infrared II (NIR-II) cell membrane-disrupting nanoflakes enables deep-penetrating and self-cascading immunotherapy for brain tumors. The nanoflakes disrupt tumor cell membranes upon NIR-II laser irradiation, facilitating the release of tumor antigens and their subsequent capture by detained DCs within the TME, initiating a potent and localized antitumor immune response (Yalamandala et al., 2024). Furthermore, for programmed immunotherapy of lung metastasis, a sophisticated cascade-responsive cell membrane-mimetic copolymer-wrapped nanoraspberry platform has been designed for coordinated elesclomol‑copper (ES-Cu) delivery. This biomimetic system achieves targeted accumulation at metastatic sites and responds to the specific pathological stimuli to release ES-Cu, which induces intense oxidative stress and cuproptosis in tumor cells, thereby triggering immunogenic cell death and potentiating a systemic antitumor immune response against metastases (Huynh et al., 2024). These pioneering studies underscore a paradigm shift toward designing intelligent nanotheranostics that can actively navigate biological barriers, interrogate the pathological microenvironment, and execute coordinated multi-modal therapies to activate sustained antitumor immunity against the most recalcitrant clinical scenarios.

## 4 Interaction between OSNs and T lymphocytes

OSNs are an innovative class of therapeutic agents designed to alleviate hypoxia in the TME (Chen et al., 2018). Their interaction with T lymphocytes is particularly critical, as T cells are central to effective anti-tumor immunity. Hypoxia not only impairs T cell functionality but also fosters a hostile environment that limits T cell recruitment, survival, and activity. OSNs address these challenges by directly enhancing T cells and indirectly reshaping the TME to restore immune balance. This section explores the mechanisms through which OSNs enhance T cell functionality, improve recruitment, and inhibit immunosuppressive elements within the TME.

### 4.1 Direct effects on T Cell functionality

**Fig. 8:**
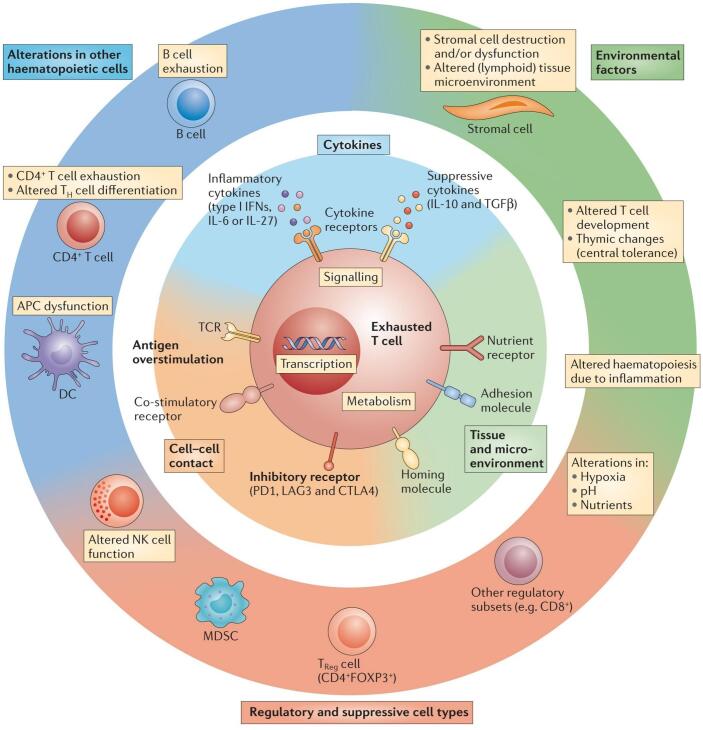
Overview of mechanisms of T cell exhaustion. (Reprint with permission from Ref. (Wherry and Kurachi, 2015).)

T cells, particularly CTLs, are vital components of the antitumor immunity. However, their function is heavily dependent on the availability of oxygen, which is severely restricted in the hypoxic TME (Fig. 8) (Wherry and Kurachi, 2015). OSNs directly enhance T cell functionality by addressing the metabolic and functional impairments induced by hypoxia. Reoxygenation of the TME through OSNs significantly improves the cytotoxic activity and proliferation of T cells. Under hypoxic conditions, CTLs exhibit reduced capacity to produce and release cytotoxic molecules such as perforin and granzyme, which are essential for tumor cell elimination. By restoring oxygen levels, OSNs enable CTLs to maintain these effector functions, leading to more effective tumor eradication. Furthermore, T cell proliferation, a process critical for expanding the pool of tumor-specific lymphocytes, is markedly enhanced under reoxygenated conditions (Colombani et al., 2021; Manaster et al., 2019). Oxygen is necessary for supporting oxidative phosphorylation in mitochondria, a key pathway that fuels the energy demands of rapidly dividing T cells (Zou et al., 2023; Guan et al., 2024). By supplying the oxygen required to sustain this metabolic process, OSNs further facilitate the expansion of the T cell population in the TME.

Different T cell subsets and states exhibit distinct metabolic dependencies and vulnerabilities to hypoxia, which in turn shape their response to OSNs. Effector and effector memory T cells, which are potent mediators of immediate cytotoxicity, rely heavily on oxidative phosphorylation for their energetic and biosynthetic demands. Consequently, they are particularly susceptible to hypoxic suppression, and their function stands to benefit most from reoxygenation by OSNs (Scharping et al., 2016; van der Windt and Pearce, 2012). In stark contrast, exhausted T cells (Tex), characterized by progressive loss of effector function and sustained expression of inhibitory receptors like PD-1, already reside in a metabolically dysfunctional state. Their mitochondria are impaired, and they exhibit a constrained metabolic profile that may alter their response to oxygen availability (Wherry and Kurachi, 2015; Bengsch et al., 2016). While hypoxia undoubtedly exacerbates exhaustion, reoxygenation alone may be insufficient to fully reverse this deeply programmed state. However, by mitigating the hypoxic drive that contributes to exhaustion and potentially lowering the threshold for checkpoint blockade, OSNs can create a more permissive environment for reinvigorating these cells, especially when combined with immunotherapy (Vodnala et al., 2019).

Furthermore, the response of engineered T cells, such as chimeric antigen receptor (CAR)-T cells, to hypoxia is a critical consideration. Their potent and rapid activation upon antigen encounter creates an immense metabolic burst, making them exceptionally vulnerable to the metabolic constraints of the hypoxic TME. This can lead to impaired persistence and premature dysfunction in solid tumors (Kawalekar et al., 2016; Gao and Chen, 2022). Therefore, OSNs may be particularly advantageous for adoptive cell therapies by providing the necessary oxygen to sustain the metabolic fitness, cytotoxicity, and longevity of these therapeutic cells, ultimately improving their efficacy against solid malignancies. This nuanced understanding underscores that the benefit of OSNs is not uniform across all T cells but is instead modulated by their differentiation and functional status.

T cell metabolism is a crucial determinant of their functionality, as distinct metabolic pathways support different phases of T cell activation and effector responses. Hypoxia forces T cells to rely on glycolysis rather than oxidative phosphorylation, leading to reduced energy generation and effector molecule synthesis. By supplying oxygen, OSNs contribute to the restoration of metabolic homeostasi, enabling T cells to shift back to oxidative phosphorylation. This metabolic reprogramming not only boosts ATP production but also facilitates the generation of ROS, which are necessary for effective T cell signaling and cytotoxic activity (Dai et al., 2024; An et al., 2020). In addition, OSN-mediated reoxygenation enhances the expression of critical cytokines such as IFN-γ, which plays a pivotal role in anti-tumor immunity (Hatfield et al., 2015; Liu et al., 2021; Čapek and T., 2021). Hypoxia suppresses IFN-γ production by inhibiting transcription factors such as STAT1 and T-bet. By alleviating hypoxia, OSNs restore the transcriptional activity of these factors, promoting cytokine secretion and strengthening T cell-mediated immune responses.

Beyond revitalizing glycolysis and oxidative phosphorylation, the impact of OSN-mediated reoxygenation likely extends to fatty acid oxidation (FAO), a metabolic pathway crucial for the long-term persistence and functionality of memory T cells. Memory T cell subsets, particularly central memory T (Tcm) cells, rely heavily on FAO for their bioenergetic and biosynthetic needs, which supports their longevity and rapid recall upon antigen re-encounter (van der Windt and Pearce, 2012; O'Sullivan et al., 2014). Hypoxia is known to suppress FAO, as this pathway requires efficient OXPHOS and is disfavored under oxygen-deprived conditions. By restoring oxygen availability, OSNs may thereby promote a metabolic shift toward increased FAO capacity in T cells. This shift could potentially enhance the generation and survival of memory T cell populations within the reoxygenated TME. The bolstering of a memory phenotype is a critical, yet often overlooked, potential benefit of OSNs, as it could contribute to durable anti-tumor immunity and reduce the risk of relapse. Therefore, the immunometabolic modulation by OSNs is not limited to boosting immediate effector functions but may also encompass the rewiring of metabolic pathways like FAO to foster the development of a robust and long-lasting T cell memory response, broadening the therapeutic scope of these nanomaterials.

### 4.2 Indirect effects via TME modulation

The hypoxic TME is exhibits multiple immunosuppressive features that hinder T cell recruitment, survival, and functionality. OSNs exert indirect effects on T cells by reshaping the TME, creating conditions that are more conducive to effective anti-tumor immunity. These effects include the modulation of chemokine gradients, normalization of vasculature, and inhibition of immunosuppressive factors. The infiltration of T cells into tumors is governed by chemokine gradients, which guide immune cells to their target locations. Hypoxia disrupts these gradients by downregulating chemokines such as CXCL9 and CXCL10, both of which are essential for recruiting CTLs (Su et al., 2024; Wang et al., 2022). OSNs help restore these chemokine gradients by alleviating hypoxia-induced suppression of chemokine production. This reoxygenation enhances the secretion of chemokines by stromal and immune cells, thereby facilitating T cells recruitment to the tumor site (Hatfield et al., 2015). Moreover, OSNs can normalize the abnormal tumor vasculature that arises due to hypoxia. Tumor vessels in hypoxic environments are often leaky, disorganized, and deficient in adhesion molecules such as ICAM-1 and VCAM-1, which are necessary for T cell extravasation (Kaur et al., 2023; Dikalova et al., 2020). By delivering oxygen to the TME, OSNs promote vascular normalization, improving endothelial cell function and restoring the expression of adhesion molecules. This vascular remodeling enhances T cell infiltration into tumors, increasing the number of effector cells that penetrate the tumor core (Castro et al., 2024; Jin et al., 2024).

Hypoxia fosters the accumulation of immunosuppressive cells, such as Tregs and MDSCs, which inhibit T cell activity. It also upregulates the expression of immune checkpoint molecules, such as PD-L1, which suppress T cell function (Ding et al., 2021; Hou et al., 2020b). OSNs counteract these effects by reoxygenating the TME, thereby reducing the prevalence and activity of immunosuppressive factors (Hatfield and Sitkovsky, 2015). For instance, OSNs decrease the recruitment and stability of Tregs by modulating the expression of chemokines such as CCL22, which mediates Treg trafficking to the tumor site (Yanamala et al., 2019; Chen et al., 2024b). They also inhibit the polarization of macrophages toward the M2-like phenotype, which is associated with immune suppression. By improving oxygenation, OSNs reduce the production of IL-10 and TGF-β by M2 macrophages, shifting the balance toward a more immunostimulatory TME. In addition, OSNs enhance the efficacy of immune checkpoint inhibitors by reducing the expression of PD-L1 on tumor and stromal cells. Hypoxia upregulates PD-L1 expression through the stabilization of HIFs. By alleviating hypoxia, OSNs inhibit HIF activity, leading to decreased PD-L1 levels and improved T cell-mediated cytotoxicity.

The hypoxic TME is characterized by increased acidity and oxidative stress, both of which impair T cell function. Acidic conditions suppress TCR signaling and cytokine production, while excessive ROS levels induce T cell apoptosis (Yan et al., 2024; Yoo et al., 2012). OSNs alleviate these conditions by reoxygenating the TME and reducing the accumulation of lactate, a byproduct of anaerobic metabolism. This restoration of physiological pH levels improves TCR signaling and cytokine secretion, enabling T cells to maintain their functionality in the TME. OSNs not only improve T cell functionality but also enhance the efficacy of various immunotherapy approaches. For example, immune checkpoint inhibitors, such as anti-PD-1 and anti-CTLA-4 antibodies, rely on the presence of functional T cells in the TME. By alleviating hypoxia, reoxygenating the TME and increasing T cell infiltration, OSNs amplify the therapeutic effects of these checkpoint inhibitors. Similarly, adoptive T cell therapies, such as CAR-T cell therapy, benefit from the improved T cell activity provided by OSNs (Hatfield et al., 2015; Hatfield and Sitkovsky, 2015; Hatfield and Sitkovsky, 2020a; Halpin-Veszeleiova et al., 2025). The reoxygenation of the TME ensures that CAR-T cells maintain their cytotoxic activity and proliferative capacity, achieving more robust tumor eradication. Additionally, OSNs can be integrated with cancer vaccines to enhance the activation of tumor-specific T cells, further boosting anti-tumor immunity (Gong et al., 2019).

A critical and emerging consideration is that the immunosuppressive cell populations targeted by OSNs are not monolithic; rather, they comprise functionally distinct subsets that may exhibit differential sensitivity to reoxygenation. For instance, within the heterogeneous Treg compartment, certain highly suppressive subsets, such as effector Tregs (eTregs), demonstrate a heightened reliance on hypoxia-stabilized HIF-1α for their stability, maintenance, and suppressive function (Abdelhafiz et al., 2021; Clambey et al., 2012). Consequently, reoxygenation mediated by OSNs could preferentially impair the survival and function of these specific, potent Treg subsets, thereby selectively dismantling a key pillar of immunosuppression without broadly affecting all regulatory circuits.

**Fig. 9:**
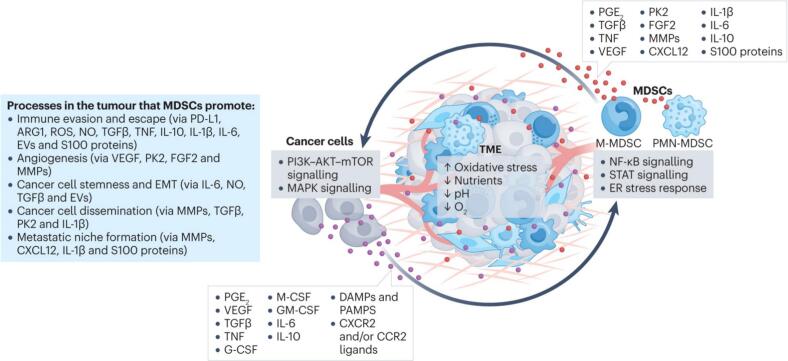
The nurturing relationship between MDSCs and tumors. (Reprint with permission from Ref. (Lasser et al., 2024).)

Similarly, the major subsets of MDSCs—polymorphonuclear MDSCs (PMN-MDSCs) and monocytic MDSCs (M-MDSCs)—may respond differently to oxygenation. Evidence suggests that PMN-MDSCs, often the more prevalent and immunosuppressive subset in many tumors, are particularly dependent on hypoxic conditions and HIF-1α signaling for their survival and activation of suppressive pathways like arginase-1 and ROS production (Noman et al., 2014; Corzo et al., 2010). Reoxygenation could therefore induce apoptosis or functional inactivation specifically in this PMN-MDSC population. In contrast, M-MDSCs might display greater plasticity, potentially undergoing differentiation into less suppressive or even immunostimulatory macrophage-like phenotypes upon oxygen availability (Fig. 9) (Lasser et al., 2024).

This concept of subset-specific susceptibility to oxygen supplementation opens promising avenues for the targeted design of OSNs. Nanomaterials could be engineered to not only reoxygenate the TME broadly but also to co-deliver agents that selectively block the survival pathways of the most oxygen-sensitive, suppressive subsets (e.g., specific HIF-1α inhibitors). Furthermore, understanding these differential responses can inform patient stratification strategies, potentially identifying those whose tumors are enriched in these oxygen-sensitive immunosuppressive subsets as most likely to benefit from OSN-based combination immunotherapies.

## 5 Enhancing tumor T cell infiltration with OSNs

TILs, particularly CTLs, play a central role in the immune response against cancer. Their infiltration into tumors is essential for immune-mediated tumor destruction and is often associated with favorable clinical outcomes. However, hypoxia in the TME poses significant barriers to T cell trafficking and recruitment. OSNs offer a promising strategy to overcome these obstacles by reoxygenating the TME, restoring chemokine gradients, and normalizing tumor vasculature to facilitate T cell infiltration. This section explores the mechanisms through which OSNs enhance T cell infiltration and reviews preclinical as well as clinical studies that demonstrate their efficacy.

### 5.1 Mechanisms of T cell trafficking and recruitment

The infiltration of T cells into tumors is a multi-step process that relies on precise chemokine gradients, functional adhesion molecules, and effective vascular permeability. Hypoxia disrupts these processes, creating a hostile environment that impairs T cell trafficking. OSNs address these challenges by restoring key molecular and normalizing structural elements within the TME.

Chemokines are small signaling proteins that create gradients to guide T cells to specific tissues, including tumors. In a healthy immune response, chemokines such as CXCL9, CXCL10, and CXCL11 bind to the CXCR3 receptor on CTLs, directing their migration toward tumor sites (Korbecki et al., 2021; Gomes-Santos et al., 2021). However, hypoxia suppresses the expression of these chemokines in both tumor cells and stromal cells, effectively disrupting the chemokine gradients necessary for T cell recruitment. The suppression of chemokine production under hypoxia is largely mediated by HIFs. HIF-1α and HIF-2α, key transcription factors activated in hypoxic conditions, downregulate chemokine expression and promote the secretion of immunosuppressive molecules such as VEGF and TGF-β, which further inhibit T cell recruitment. As a result, the TME becomes deficient in the signals required to attract T cells, thereby limiting their infiltration.

In addition to chemokine gradients, T cell infiltration requires functional adhesion molecules, such asICAM-1 andVCAM-1, which mediate the attachment of T cells to the endothelium. Hypoxia-induced abnormalities in tumor vasculature reduce the expression of these adhesion molecules, impairing the ability of T cells to extravasate from blood vessels into tumor tissue. Tumor blood vessels in the hypoxic TME are also structurally defective, characterized by excessive leakiness, disorganization, and poor perfusion. These vascular abnormalities create physical barriers to T cell trafficking, further limiting their access to the tumor interior.

**Fig. 10:**
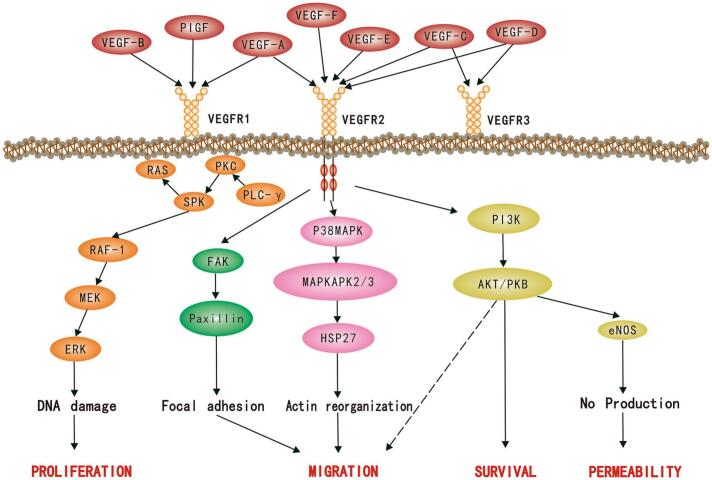
VEGF signaling pathway induces the process of angiogenesis. (Reprint with permission from Ref. (Teng et al., 2024).)

OSNs play a crucial role in addressing the above challenges by reoxygenating the TME and restoring the molecular and structural components necessary for T cell trafficking. By delivering or generating oxygen in the hypoxic regions of tumors, OSNs alleviate the effects of HIFs, thereby upregulating the expression of chemokines such as CXCL9 and CXCL10. This restoration of chemokine gradients promotes the recruitment of CTLs to the tumor site. Additionally, OSNs promote the normalization of tumor vasculature, improving its structure and function. Oxygenation facilitates the expression of adhesion molecules like ICAM-1 and VCAM-1 on endothelial cells, enabling effective interactions between T cells and the tumor vasculature. Nanomaterials loaded with agents such as VEGF inhibitors or anti-angiogenic molecules further aid in vascular remodeling, creating a more organized and perfused vasculature that supports T cell extravasation (Fig. 10) (Teng et al., 2024; Gao et al., 2023). Some OSNs are engineered to actively interact with immune cells or endothelial cells to enhance their functionality. For example, nanomaterials conjugated with targeting ligands or antibodies can selectively bind to immune cell receptors, amplifying their recruitment and retention in the tumor. Others incorporate payloads that release cytokines or growth factors, which further stimulate T cell migration and infiltration.

### 5.2 Examples of clinical studies

A growing body of evidence from preclinical and clinical studies demonstrates that OSNs can markedly enhance T cell infiltration into tumors. These studies highlight the potential of OSNs to overcome hypoxia-induced barriers and improve the efficacy of cancer immunotherapy.

A study using catalase-loaded nanoparticles demonstrated their ability to decompose H₂O₂ into oxygen, alleviating hypoxia in the TME (Heble et al., 2021; Qi et al., 2011). The reoxygenation resulted in increased production of CXCL9 and CXCL10, leading to enhanced CTL infiltration. In a murine model of melanoma, tumors treated with catalase-loaded nanoparticles exhibited significantly higher levels of T cell infiltration compared to untreated controls, resulting in reduced tumor growth (Hei et al., 2020).

**Fig. 11:**
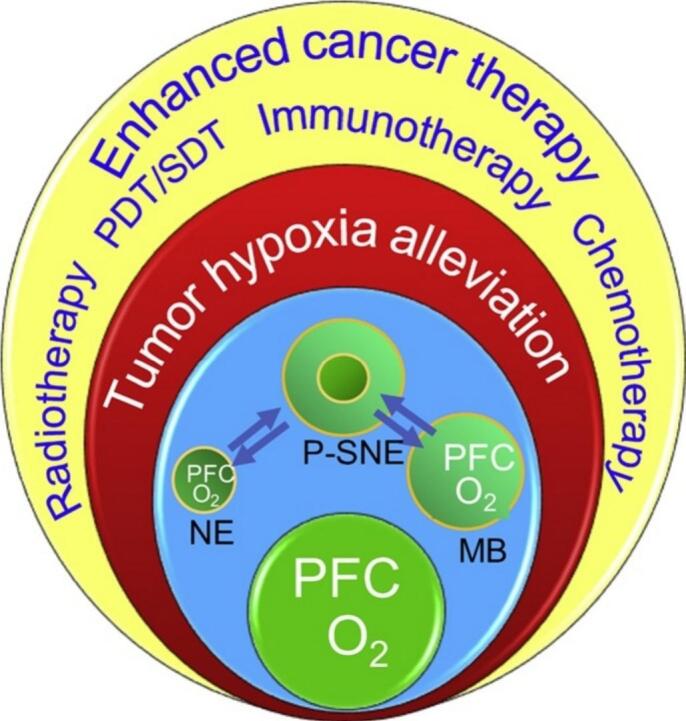
Alleviating tumor hypoxia with perfluorocarbon-based oxygen carriers. (Reprint with permission from Ref. (Krafft, 2020).)

PFC-based nanoparticles have been shown to deliver oxygen directly to hypoxic tumors, restoring oxygen levels and normalizing vasculature. In a breast cancer model,treatment with PFC nanoparticles increased the expression of adhesion molecules on endothelial cells and improved T cell extravasation (Fig. 11) (Krafft, 2020). The combination of PFC nanoparticles with immune checkpoint inhibitors further amplified T cell-mediated tumor destruction.

Hemoglobin-based nanomaterials, designed to transport and release oxygen in the TME, have shown promising results in enhancing T cell infiltration (Jia et al., 2016; Jansman and Hosta-Rigau, 2018). In a mouse model of colorectal cancer, hemoglobin-mimicking nanoparticles increased T cell recruitment to tumors by restoring chemokine gradients and reducing immunosuppressive cytokine levels (Jansman et al., 2020). The nanoparticles also synergized with PD-1 blockade therapy, improving overall survival in treated mice.

MOFs engineered to release oxygen and inhibit HIF activity have demonstrated efficacy in promoting T cell infiltration in hypoxic tumors. In a preclinical lung cancer model, MOFs treatment increased the density of TILs and improved the response to adoptive T cell therapy. The study also highlighted the ability of MOFs to reduce the accumulation of Tregs, further enhancing anti-tumor immunity (Ni et al., 2020b).

### 5.3 Examples of preclinical studies

**Fig. 12:**
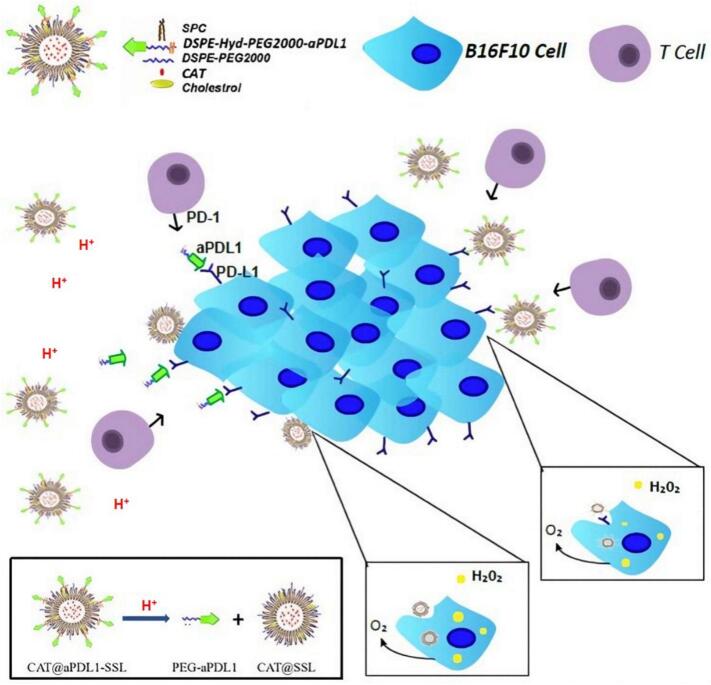
The schematic diagram of the structure and activating mechanism of CAT@aPDL1-SSL. (Reprint with permission from Ref. (Hei et al., 2020).)

Early-phase clinical trials have begun to explore the use of oxygen-generating nanomaterials in combination with checkpoint inhibitors. In one trial, catalase-loaded nanoparticles combined with anti-PD-1 therapy for advanced melanoma led to enhanced T cell infiltration and improved overall response rates compared to anti-PD-1 monotherapy (Fig. 12) (Hei et al., 2020). Clinical studies evaluating PFC nanoparticles in patients with hypoxia-associated cancers, such as head and neck squamous cell carcinoma, have shown promising results (Abdelgwad et al., 2022; Han et al., 2020). Tumors treated with PFC nanoparticles exhibited reduced hypoxia, increased TIL density, and enhanced sensitivity to radiation therapy and immunotherapy. Hemoglobin-based and polymeric nanomaterials have been evaluated for safety and efficacy in several clinical settings. These studies have confirmed their ability to deliver oxygen effectively to tumors, improve vascular function, and enhance T cell recruitment without significant toxicity.

## 6 Enhancing T cell responses in the tumor

Effective anti-tumor immunity hinges on the functionality and persistence of T cells within the TME. Hypoxia, a hallmark of the TME, profoundly suppresses T cell responses, limiting their cytotoxic activity and impairing therapeutic interventions such as immune checkpoint inhibitors and adoptive cell therapies. OSNs have emerged as a promising solution to address these challenges, not only by alleviating hypoxia but also by synergizing with immunotherapies, reducing immunosuppression, and enhancing immune activation. This section discusses how OSNs enhance T cell responses in the TME, including their role in boosting immune activation, mitigating immunosuppressive factors, and serving as components of combination strategies.

### 6.1 Boosting immune activation

OSNs enhance T cell responses by directly restoring the functionality of T cells within the hypoxic TME and by synergizing with immunotherapeutic approaches. Immune checkpoint inhibitors, such as anti-PD-1, anti-PD-L1, and anti-CTLA-4 antibodies, have revolutionized cancer treatment by reactivating exhausted T cells. However, their therapeutic efficacy is often limited in hypoxic tumors due to reduced T cell infiltration and activity. OSNs address these limitations by reoxygenating the TME, which enhances T cell metabolic fitness and promotes effector function. Studies have demonstrated that oxygenated environments amplify the efficacy of immune checkpoint inhibitors by increasing T cell proliferation, cytokine production, and cytotoxic activity (Baldwin et al., 2024; Li et al., 2022). For instance, catalase-loaded nanoparticles used alongside anti-PD-1 therapy have been shown to significantly enhance tumor regression in preclinical models of melanoma (Hei et al., 2020).

**Fig. 13:**
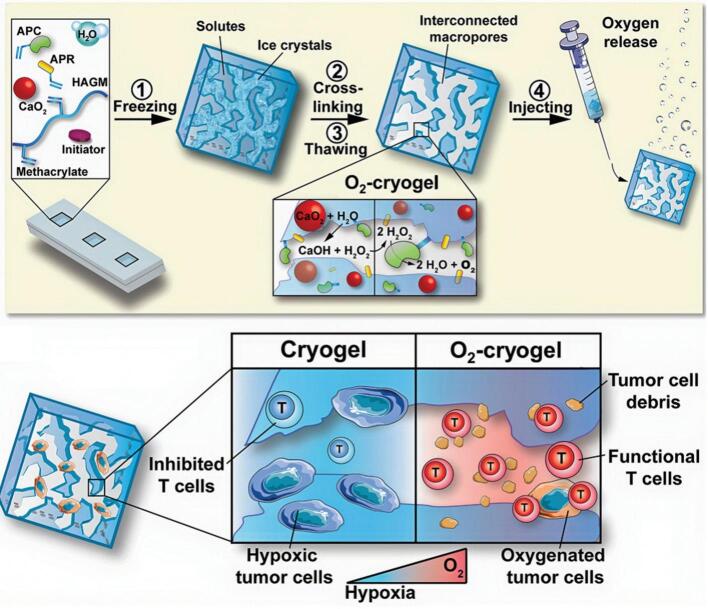
The schematic diagram of the structure and activating mechanism of CAT@aPDL1-SSL. (Reprint with permission from Ref. (Colombani et al., 2021).)

CAR-T cell therapy involves engineering T cells to specifically target tumor-associated antigens, offering a potent therapeutic option for cancers. However, the hypoxic TME limits the efficacy of CAR-T cells by impairing their persistence and cytotoxicity. OSNs improve the metabolic conditions within tumors, enabling CAR-T cells to sustain their activity. In addition, OSNs can be engineered to deliver adjuvants or co-stimulatory molecules that further boost CAR-T cell expansion and functionality (Lybaert et al., 2018; Li et al., 2020). Preclinical studies have shown that pairing OSNs with CAR-T cells enhances tumor infiltration and prolongs CAR-T cell survival, improving anti-tumor efficacy. Cancer vaccines aim to prime the immune system by presenting tumor-associated antigens to T cells, stimulating a robust anti-tumor response. However, the immunosuppressive and hypoxic TME often limits the efficacy of these vaccines. OSNs can enhance vaccine-induced immunity by reprogramming the TME to be more conducive to immune activation (Hatfield and Sitkovsky, 2015; Lybaert et al., 2018; Yang et al., 2023b). For example, oxygen-generating nanomaterials used in conjunction with peptide-based vaccines have been shown to improve T cell activation and effector function, leading to more effective tumor clearance in preclinical models (Cheng et al., 2018). This concept is further powerfully illustrated by the pioneering development of oxygen-generating cryogels, which represent a seminal demonstration of biomaterial-based oxygen delivery for immunotherapy. These cryogels were shown to sustainably release oxygen within the TME, effectively reversing hypoxia and restoring T cell-mediated cytotoxicity, thereby providing a robust platform to enhance the efficacy of cell-based therapies in hypoxic tumors (Fig. 13) (Colombani et al., 2021).

The synergy between OSNs and advanced immunotherapies is moving from concept toward clinical reality, with several pioneering examples underscoring the potential of co-design strategies. In the realm of CAR-T cell therapy, efforts are underway to engineer “next-generation” constructs that are inherently more resistant to the suppressive TME. For instance, CAR-T cells engineered to overexpress catalase (CAT-CAR-T cells) represent a direct fusion of biological oxygen generation and cell therapy, designed to self-modulate their immediate hypoxic microenvironment and maintain potency in solid tumors (Mangal et al., 2021). The combination of such innovative CAR designs with externally administered OSNs could offer a multi-layered approach to overcome hypoxia. Similarly, in cancer vaccines, nanotechnology platforms are being co-designed for dual antigen and oxygen delivery. An exemplary strategy involves the use of nanoscale nMOFs that not only serve as oxygen reservoirs but also as carriers for tumor neoantigens or immunostimulatory agents, creating a potent in situ vaccine that primes and sustains T cell responses in a reoxygenated niche (Ni et al., 2020b).

These concrete examples and ongoing clinical efforts highlight the transition from high-level concept to engineered reality, providing a robust framework for developing the next wave of OSN-combination therapies.

### 6.2 Reducing immunosuppression

**Fig. 14:**
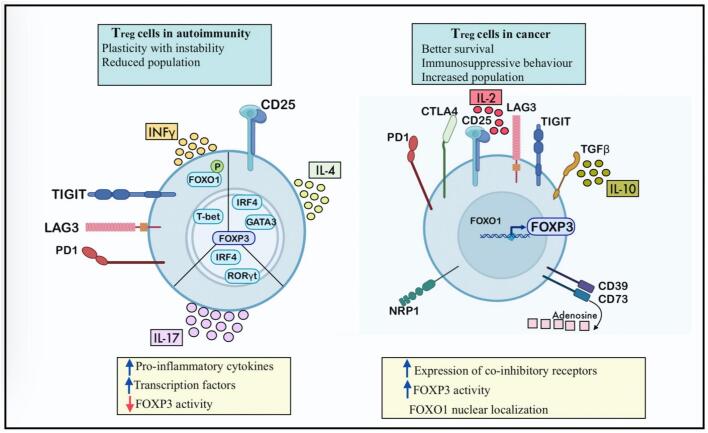
Characterization of T regulatory cells. (Reprint with permission from Ref. (Sureka and Zaheer, 2025).)

Hypoxia in the TME promotes the accumulation of immunosuppressive cells, such as MDSCs and Tregs, which inhibit T cell responses and foster tumor progression. OSNs mitigate these effects by reoxygenating the TME and reducing the prevalence and activity of these immunosuppressive populations. MDSCs constitute a heterogeneous population of immature myeloid cells that suppress T cell activity through various mechanisms, including the depletion of essential nutrients (e.g., arginine) and the production of immunosuppressive molecules such as ROS and nitric oxide (NO). Hypoxia amplifies the recruitment and activity of MDSCs by upregulating HIFs and immunosuppressive cytokines such as VEGF and GM-CSF (Zhu et al., 2024b). OSNs disrupt these pathways by reducing HIF-mediated signaling, thereby decreasing the accumulation and suppressive activity of MDSCs (Li et al., 2024b). Preclinical studies employing OSNs have demonstrated significant reductions in MDSC infiltration, correlating with enhanced T cell functionality and tumor regression. Tregs play a critical role in maintaining immune tolerance but are co-opted by tumors to suppress anti-tumor immunity (Sureka and Zaheer, 2025). Hypoxia promotes Treg recruitment and stability by upregulating chemokines such as CCL22 and enhancing FoxP3 expression, a transcription factor critical for Treg function (Golzari-Sorkheh and Zúñiga-Pflücker, 2023). OSNs reduce Treg prevalence in the TME by reoxygenating hypoxic regions, thereby disrupting chemokine signaling and Treg stability. Additionally, the improved oxygenation facilitates a shift in the cytokine milieu from an immunosuppressive (IL-10, TGF-β) to an immunostimulatory (IFN-γ, IL-12) profile, further reducing Treg-mediated suppression (Fig. 14) (Sureka and Zaheer, 2025).

### 6.3 Combination strategies

The multifunctional capabilities of OSNs render them ideal candidates for integration into combination therapies. By alleviating hypoxia and simultaneously enhancing immune activation, OSNs synergize with other therapeutic approaches, amplifying their efficacy. Cytokine-based therapies, such as interleukin-2 (IL-2) and interleukin-15 (IL-15), are designed to stimulate T cell proliferation and activity (Yang and A., 2020). However, their efficacy is often limited by the immunosuppressive effects of the hypoxic TME. OSNs complement cytokine therapies by creating a reoxygenated environment that enhances cytokine signaling.

Adoptive T cell therapies, including TIL therapy, involve the expansion and reinfusion of T cells to target tumors (Met et al., 2019). However, the hypoxic TME limits the effectiveness of these therapies by suppressing T cell infiltration and functionality (Hatfield et al., 2015). OSNs can enhance the therapeutic potential of adoptive T cell therapies by reprogramming the TME to support T cell activity. Studies have shown that OSNs paired with adoptive T cell therapy increase T cell infiltration, reduce tumor growth, and extend survival in murine models (Hatfield et al., 2015). Radiotherapy and chemotherapy are commonly used in cancer treatment but are often less effective in hypoxic tumors. OSNs enhance the efficacy of these therapies by reoxygenating tumors, thereby increasing tumor sensitivity to radiation-induced DNA damage and chemotherapeutic agents. Additionally, the improved immune activation facilitated by OSNs can synergize with the immune-boosting effects of radiotherapy and chemotherapy, resulting in more comprehensive tumor eradication.

OSNs offer a transformative approach to enhancing T cell responses in the TME. By alleviating hypoxia, OSNs restore T cell functionality, amplify the efficacy of immunotherapies, and mitigate immunosuppressive factors. Their ability to synergize with immune checkpoint inhibitors, CAR-T cells, and cancer vaccines highlights their versatility as therapeutic agents. Additionally, OSNs can deplete MDSCs and Tregs populations, further strengthening anti-tumor immunity. As research continues to optimize the design and application of OSNs, their integration into combination strategies promises to improve therapeutic outcomes and expand the possibilities for effective cancer immunotherapy.

## 7 Challenges and opportunities

OSNs represent a promising avenue for addressing the hypoxia-driven challenges of the TME. By delivering or generating oxygen, these materials improve T cell functionality, enhance immune cell infiltration, and boost overall immune responses. Despite their potential, OSNs still face several technical and biological challenges that hinder their translation into widespread clinical use. Concurrently, the field offers substantial opportunities for innovative research to further enhance the design, optimize the functionality, and expand the application of these materials. This section explores the key challenges associated with OSNs and outlines the opportunities for future development.

### 7.1 Technical challenges

The translation of OSNs from the laboratory to clinical settings is hindered by several technical obstacles, including delivery, stability, safety, and potential off-target effects. The effectiveness of OSNs depends on their ability to supply or generate oxygen precisely within hypoxic tumor regions. However, achieving targeted delivery remains a significant challenge. Nanomaterials rely on the enhanced permeability and retention (EPR) effect to accumulate in tumors, but the inherent heterogeneity of tumor vasculature and microenvironments often limits their penetration and distribution. Factors such as poor perfusion and dense ECM components further restrict the intratumoral transport of OSNs, reducing their efficacy. Another concern is the stability of nanomaterials in the bloodstream. OSNs can face rapid degradation or clearance by the mononuclear phagocyte system (MPS), particularly by macrophages in the liver and spleen. To address this, surface modifications have been explored, such as polyethylene glycol (PEG) coatings, to enhance stability and prolong circulation time. However, these coatings may trigger immune responses, known as the “PEG dilemma,” which limits their widespread applicability (Kang et al., 2022; Dabagh et al., 2023).

Nanomaterials interact with a variety of biological systems, raising concerns about potential off-target effects and toxicity. For instance, MOFs and certain catalytic nanoparticles may release toxic ions or generate excessive ROS beyond therapeutic levels, causing oxidative damage to healthy tissues. Similarly, PFC-based nanoparticles, while effective as oxygen carriers, can accumulate in organs such as the liver and lungs, potentially leading to long-term toxicity (Day and Sletten, 2021; Han et al., 2021b). Furthermore, the biodistribution and clearance of OSNs remain incompletely understood. Non-biodegradable or poorly degradable materials can persist in the body, posing risks of chronic toxicity. Ensuring the biocompatibility and safe elimination of OSNs is crucial for their clinical translation (Lee et al., 2017; Huang et al., 2023).

Beyond the general concerns of stability and off-target effects, a more thorough evaluation of specific biocompatibility challenges is warranted. Firstly, the therapeutic generation of ROS is a double-edged sword. While controlled ROS production is the goal of therapies like PDT and can stimulate immune activation, ROS overproduction can lead to severe cytotoxicity, not only to tumor cells but also to healthy tissues and infiltrating immune cells, potentially causing unintended tissue damage and inflammation (Lee et al., 2013; Fang et al., 2009). This necessitates the precise design of nanomaterials with controllable ROS generation kinetics.

Secondly, the issue of long-term biodistribution and accumulation requires careful scrutiny. For instance, certain non-biodegradable or slowly degrading nanomaterials, such as some MOFs or PFCs, may accumulate in the MPS organs, particularly the liver and spleen, after repeated administration (Day and Sletten, 2021; Bollhorst et al., 2017). The long-term consequences of this accumulation, including potential chronic inflammation, organ toxicity, or unforeseen immune modulation, remain a critical unanswered question that must be addressed through comprehensive long-term in vivo studies.

Finally, the very goal of enhancing immune responses carries the inherent risk of immune overactivation. By reoxygenating the TME and potentiating T cell functionality, OSNs could theoretically contribute to a hyperinflammatory state. In a worst-case scenario, this could precipitate a cytokine release syndrome (CRS)-like event, especially when combined with potent immunotherapies like CAR-T cells or immune checkpoint inhibitors (Shimabukuro-Vornhagen et al., 2018; Morris et al., 2022). The potential for triggering autoimmune reactions against self-antigens also warrants consideration. Therefore, the development of strategies to precisely modulate the immune response—avoiding both suppression and excessive activation—is paramount for the safe clinical translation of OSNs.

### 7.2 Unresolved challenges and controversies

While the potential of OSNs is considerable, a balanced perspective necessitates a critical examination of several unresolved biological and clinical controversies that could impede their translation.

Firstly, the role of ROS presents a significant paradox. While many OSN strategies, particularly those involving catalytic nanomaterials or PDT, rely on or generate ROS for therapeutic effect, the risk of ROS-associated toxicity remains a serious concern (Lee et al., 2013; Sies and Jones, 2020). Off-target or excessive ROS production can inflict severe damage on lipids, proteins, and DNA, not only in tumor cells but also in healthy tissues and, crucially, in the very immune cells (e.g., T lymphocytes) that OSNs aim to empower. This oxidative stress can lead to non-specific inflammation, exacerbate tissue damage, and even induce apoptosis in effector immune cells, potentially counteracting the intended immunostimulatory benefits. The precise control over ROS generation to maintain a therapeutic window—sufficient for tumor cell killing without harming the immune response—is a major unresolved challenge.

Secondly, the variable efficacy of adoptive cell therapies, notably CAR-T cells, in solid tumors remains a formidable hurdle that OSNs alone may not overcome. While hypoxia is a key barrier, solid tumors present additional obstacles including physical barriers (dense ECM), inadequate tumor trafficking, antigen heterogeneity, and an immunosuppressive milieu rich in cytokines like TGF-β (Sterner and Sterner, 2021; Martinez and Moon, 2019). The optimistic assumption that reoxygenation alone will universally unlock CAR-T cell efficacy in solid tumors is not fully supported by clinical evidence. The response is likely to be highly variable, dependent on the specific CAR construct, tumor type, and the complex interplay of other immunosuppressive factors beyond hypoxia. Therefore, OSNs should be viewed as a potent component of a combination strategy rather than a standalone solution for CAR-T therapy in solid tumors.

Finally, the strategy of enhancing immune responses inherently carries the underappreciated risk of immune overstimulation. The potentiation of T cell functionality and cytokine production by OSNs, especially when combined with powerful immunotherapies like immune checkpoint inhibitors (ICIs) or CAR-T cells, could theoretically precipitate a systemic hyperinflammatory state. This could manifest as cytokine release syndrome (CRS) or immune-related adverse events (irAEs), which are serious and potentially life-threatening complications (Shimabukuro-Vornhagen et al., 2018). The possibility of triggering autoimmune reactions against self-antigens released during tumor cell death also warrants careful consideration. The field currently lacks predictive biomarkers to identify patients at high risk for such adverse events following OSN-augmented therapy, representing a significant knowledge gap in safely managing these potent combinations.

Acknowledging these controversies is not to diminish the promise of OSNs but to provide a more realistic framework for their development. Addressing these issues will require rigorous preclinical modeling, careful patient selection in clinical trials, and the development of sophisticated safety strategies, such as biomarkers for monitoring immune activation and controllable “safety switch” mechanisms within nanomaterial design.

### 7.3 Opportunities for future research

While technical challenges persist, they also open the door for innovation and development. Advances in materials science, molecular engineering, and personalized medicine present exciting opportunities to optimize OSNs for cancer immunotherapy. The future of OSNs lies in their ability to perform multiple functions simultaneously. Multifunctional nanomaterials that combine oxygen delivery with immune-stimulatory capabilities hold great promise for overcoming the immunosuppressive TME. For example, nanomaterials can be engineered to deliver oxygen while releasing immunomodulatory agents such as cytokines, immune checkpoint inhibitors, or tumor antigens. These integrated systems could not only alleviate hypoxia but also directly activate T cells or enhance antigen presentation, amplifying anti-tumor immunity. Recent advances in stimuli-responsive nanomaterials provide additional opportunities for precision delivery. These materials can be designed to release their therapeutic payloads in response to specific stimuli within the TME, such as acidic pH, high ROS levels, or hypoxia. This ensures that the therapeutic effects are localized to the tumor, minimizing off-target toxicity.

A critical consideration for the clinical translation of OSNs is the profound tumor heterogeneity across patients and even within a single tumor mass. This variability can significantly influence OSN performance and must be addressed through personalized approaches. Key aspects of heterogeneity include:

Variable Oxygen Gradients: Tumors exhibit highly heterogeneous and dynamic hypoxia patterns. The efficacy of OSNs is contingent upon their ability to penetrate and function within these specific hypoxic niches. A uniformly administered OSN may fail to reach or adequately reoxygenate the most severely hypoxic and treatment-resistant regions, which are often the most immunosuppressive (Trédan et al., 2007; Horsman and Van Der Kogei, 2009).

Diverse Immune Cell Composition: The immune contexture of the TME (e.g., the ratio of CTLs to Tregs, the presence of MDSCs, and macrophage polarization) varies greatly between cancer types and individual patients. An OSN that successfully reoxygenates a “T cell-inflamed” tumor may boost immunotherapy, but the same strategy might have limited effect in a “T cell-desert” or heavily myeloid-infiltrated tumor, where the fundamental immune components necessary for a response are absent or suppressed (Binnewies et al., 2018; Galon and Bruni, 2019).

ECM Density: A dense, fibrotic ECM, common in pancreatic or breast cancers, presents a major physical barrier to nanoparticle penetration and distribution. The high interstitial fluid pressure in such tumors can impede the convective transport of OSNs, preventing them from reaching their intended targets far from blood vessels (Jain, 2013; Provenzano and Hingorani, 2013).

Tumors are highly heterogeneous, with variations in hypoxic levels, immune profiles, and vascularization across patients and even within the same tumor. This heterogeneity necessitates personalized approaches to nanomedicine. Future research can focus on tailoring OSNs to the specific characteristics of an individual's tumor. Advanced imaging and biomarker analysis techniques can play a pivotal role in personalizing OSNs design. For example, hypoxia imaging techniques such as positron emission tomography (PET) or magnetic resonance imaging (MRI) can identify regions of severe hypoxia, guiding the administration of OSNs. Similarly, genetic and proteomic profiling of tumors can inform the selection of immune-stimulatory payloads or targeting ligands to enhance therapeutic efficacy.

The integration of OSNs with other cutting-edge therapies offers significant opportunities for improving treatment outcomes. For example, OSNs can be combined with CAR-T cell therapy or adoptive T cell transfer to enhance T cell functionality within the TME. By alleviating hypoxia, OSNs ensure that engineered T cells maintain their cytotoxic activity and persistence, improving therapeutic efficacy. Additionally, OSNs can be paired with cancer vaccines to create a more favorable environment for vaccine-induced immune responses. Reoxygenation of the TME enhances antigen presentation and T cell activation, boosting the effectiveness of vaccines in inducing durable immunity. To address concerns about toxicity and clearance, future research should prioritize the design of biodegradable and biocompatible OSNs. Materials such as natural polymers (e.g., chitosan, hyaluronic acid) and lipids offer promising alternatives to synthetic materials. These biodegradable systems break down into non-toxic byproducts, reducing the risk of long-term accumulation and chronic toxicity (Feng et al., 2022; Xie et al., 2019). Innovations in self-assembling nanomaterials, which form structures in situ and degrade after delivering their payloads, also hold great potential. These systems provide high therapeutic efficacy while ensuring safety and minimal environmental impact within the body.

### 7.4 Combining challenges and opportunities into clinical translation

While the majority of OSN research remains in the preclinical domain, several pioneering platforms have advanced through critical IND-enabling studies, offering valuable insights into the clinical translation pathway. Highlighting these front-runners provides a realistic gauge of the field's maturity. For instance, dodecafluoropentane emulsion (DDFPe/NVX-108), a PFC-based oxygen carrier, has successfully completed GLP toxicology studies. It is designed to transiently increase tumor oxygenation and has been evaluated in clinical trials in combination with radiotherapy for glioblastoma (NCT02189109), demonstrating the feasibility of systemically administered oxygen-delivery nanomaterials (Lickliter et al., n.d.; Unger et al., n.d.; Lickliter et al., 2023). Another notable example is Hafnium oxide nanoparticles (NBTXR3), which, although not a classical oxygen supplier, function as radio-enhancers that potentiate radiation-induced DNA damage and subsequent immunogenic cell death. Their activation is significantly amplified under hypoxia, and they have received regulatory approval in Europe for locally advanced soft tissue sarcoma, with extensive clinical trials underway in other cancer types, including head and neck cancer (NCT04892173) (Bonvalot et al., 2019; Le Tourneau et al., n.d.). The progression of these platforms underscores that the clinical translation of nanomaterial-based therapies for modulating the TME is not only possible but already in progress. Their development path—addressing scalable manufacturing, pharmacokinetics, and safety profiles under stringent GLP conditions—serves as a critical blueprint for the next generation of OSNs aiming for clinical application.

The clinical translation of OSNs requires a balanced approach that addresses technical challenges while leveraging opportunities for innovation. Rigorous preclinical evaluation is essential to assess the safety, efficacy, and pharmacokinetics of OSNs. Animal models that closely mimic the human TME can provide valuable insights into the behavior of nanomaterials in vivo. Collaborative efforts among researchers, clinicians, and regulatory agencies are also critical for advancing OSNs into clinical trials. Establishing standardized protocols for the synthesis, characterization, and testing of OSNs will ensure consistency and reliability in their application. Furthermore, the incorporation of OSNs into combination therapy regimens, such as immune checkpoint inhibitors or radiation therapy, could accelerate clinical adoption by demonstrating additive or synergistic effects in enhancing patient outcomes.

OSNs hold immense potential for transforming cancer immunotherapy by addressing hypoxia-induced barriers in the TME. However, their successful translation into clinical practice depends on overcoming key challenges related to delivery, stability, safety, and toxicity. At the same time, advances in multifunctional nanomaterial design, personalized medicine, and integration with emerging therapies present exciting opportunities to optimize OSNs for more effective and tailored treatments. By addressing these challenges through innovative research and collaboration, OSNs can become a cornerstone of next-generation cancer therapies. Their ability to alleviate hypoxia, modulate the immune response, and synergize with other treatments positions them as a powerful tool in the fight against cancer. With continued progress, OSNs offer the promise of improving survival and quality of life for patients with hypoxia-associated tumors.

## 8 Conclusion

The intersection of nanotechnology and immunology has unveiled transformative possibilities for cancer therapy, with OSNs standing at the forefront of this innovation. Hypoxia, a hallmark of the TME, creates a formidable barrier to effective cancer treatment by impairing T cell infiltration, suppressing immune responses, and fostering resistance to conventional and immunotherapies. OSNs address these challenges through their ability to alleviate hypoxia, restore T cell functionality, and reprogram the TME. This concluding section summarizes the findings on the critical role of OSNs in overcoming hypoxia-driven immune suppression and discusses the future perspectives for advancing this technology in cancer immunotherapy.

OSNs represent a promising strategy to tackle one of the most intractable challenges in cancer therapy: hypoxia. By delivering or generating oxygen within the hypoxic TME, OSNs reoxygenate tumors and reverse the immune-suppressive effects of low oxygen levels. This reoxygenation enhances the recruitment, activation, and functionality of T lymphocytes, which are essential for effective anti-tumor immunity. Hypoxia significantly impairs T cell responses by suppressing chemokine gradients, reducing vascular adhesion molecule expression, and promoting the accumulation of immunosuppressive cell populations such as Tregs and MDSCs. OSNs address these barriers by restoring oxygen levels, which reverses the metabolic and functional impairments caused by hypoxia. This results in improved T cell infiltration, enhanced cytotoxicity, and increased cytokine production within the TME.

The integration of OSNs into cancer immunotherapy regimens holds the potential to revolutionize treatment outcomes. By alleviating hypoxia, these nanomaterials amplify the efficacy of immune checkpoint inhibitors, adoptive T cell therapies, and cancer vaccines. They also normalize tumor vasculature, facilitating the extravasation and infiltration of T cells into tumor tissue. Furthermore, the multifunctionality of OSNs—combining oxygen delivery with the release of immunomodulatory agents or synergizing with photodynamic and radiation therapies—positions them as versatile tools in the fight against cancer. Preclinical studies have provided compelling evidence of the efficacy of OSNs in improving T cell infiltration and activity. For example, catalase-loaded nanoparticles have been shown to generate oxygen in situ, boosting the immune response in murine models of melanoma and lung cancer. Clinical trials, although in their early stages, have demonstrated the safety and potential of OSNs in combination with immune checkpoint inhibitors and radiation therapy. These findings underscore the translational potential of OSNs in addressing hypoxia-associated tumors.

The successful implementation of OSNs in clinical oncology will require integrative research efforts that span nanotechnology, immunology, and clinical practice. Progress in this field hinges on addressing current challenges and exploring new opportunities for innovation and application. Future research should focus on optimizing the design of OSNs to enhance their delivery, stability, and functionality. Multifunctional nanomaterials that integrate oxygen delivery with immune-stimulatory payloads, such as cytokines or checkpoint inhibitors, hold significant promise. Stimuli-responsive systems that release their therapeutic cargo in response to TME-specific conditions (e.g., pH, hypoxia, or ROS levels) can further enhance the precision and efficacy of OSNs. Additionally, the development of biodegradable and biocompatible materials should be prioritized to minimize toxicity and ensure safe clearance from the body.

Tumor heterogeneity presents a major challenge in cancer therapy, necessitating personalized approaches to OSNs application. Advanced imaging techniques, such as hypoxia PET scans, alongside genomic or proteomic profiling can guide the customization of OSNs design and administration. For example, personalized OSNs can be tailored to the unique hypoxic profile, immune landscape, and molecular characteristics of an individual's tumor, maximizing therapeutic outcomes. The integration of OSNs with cutting-edge cancer therapies offers exciting opportunities for combination treatments. For instance, pairing OSNs with CAR-T cell therapy can enhance the persistence and activity of engineered T cells in the hypoxic TME. Similarly, OSNs can be used in conjunction with cancer vaccines to create a more favorable environment for antigen presentation and T cell activation. Exploring these synergistic strategies can unlock noveltherapeutic avenues and improve the efficacy of existing treatments.

To translate OSNs into routine clinical use, both technical and regulatory challenges must be addressed. Rigorous preclinical testing is needed to evaluate the safety, biodistribution, and pharmacokinetics of these materials. Standardized manufacturing processes and quality control protocols will be essential to ensure consistency and reliability. Collaborative efforts among researchers, clinicians, and regulatory agencies can facilitate the development of guidelines for clinical trials and approval pathways for OSNs. While the primary focus of OSNs has been on cancer therapy, their potential applications could extend to other hypoxia-related diseases, such as ischemic disorders, chronic inflammatory conditions, and infectious diseases. Exploring these broader applications can expand the impact of OSN technology and provide solutions for a wide range of medical challenges.

OSNs have emerged as a groundbreaking innovation in the field of cancer immunotherapy, addressing the pervasive challenge of hypoxia in the TME. By restoring oxygen levels, these materials enhance T cell functionality, improve infiltration, and reprogram the TME to support immune activation. Their potential to synergize with existing therapies, such as immune checkpoint inhibitors and CAR-T cell therapy, highlights their versatility and transformative potential. The future of OSNs lies in integrative research efforts that combine advancements in nanotechnology with insights from immunology and clinical oncology. Personalized approaches, innovative material design, and strategic combinations with emerging therapies will pave the way for the successful translation of OSNs into clinical practice. As these efforts progress, OSNs are poised to redefine the landscape of cancer treatment, offering new hope for patients with hypoxia-associated tumors and setting the stage for the next generation of precision medicine.

## CRediT authorship contribution statement

**Shuo Xiang:** Conceptualization, Investigation, Visualization, Writing – original draft. **Hui Zhan:** Writing – original draft, Visualization, Investigation, Formal analysis, Data curation. **Jimin Zhan:** Writing – review & editing, Supervision, Conceptualization. **Xin Li:** Writing – review & editing, Supervision, Project administration, Funding acquisition, Conceptualization. **Xiaoji Lin:** Conceptualization, Investigation, Project administration, Supervision, Validation, Writing – review & editing. **Wenjie Sun:** Conceptualization, Investigation, Project administration, Visualization, Writing – review & editing.

## Declaration of competing interest

The authors declare no conflict of interest.

## Data Availability

Data will be made available on request.
